# A novel bi-domain plant defensin MtDef5 with potent broad-spectrum antifungal activity binds to multiple phospholipids and forms oligomers

**DOI:** 10.1038/s41598-017-16508-w

**Published:** 2017-11-23

**Authors:** Kazi T. Islam, Siva L. S. Velivelli, R. Howard Berg, Blake Oakley, Dilip M. Shah

**Affiliations:** 1 Donald Danforth Plant Science Center, St Louis, MO 63132 USA; 2 Present Address: Department of Plant Pathology, University of Georgia, Athens, GA 30602 USA

## Abstract

Defensins are cysteine-rich cationic antimicrobial peptides contributing to the innate immunity in plants. A unique gene encoding a highly cationic bi-domain defensin MtDef5 has been identified in a model legume *Medicago truncatula*. MtDef5 consists of two defensin domains of 50 amino acids each linked by a 7-amino acid peptide. It exhibits broad-spectrum antifungal activity against filamentous fungi at submicromolar concentrations. It rapidly permeabilizes the plasma membrane of the ascomycete fungi *Fusarium graminearum* and *Neurospora crassa* and induces accumulation of reactive oxygen species. It is internalized by these fungi, but uses spatially distinct modes of entry into these fungi. It co-localizes with cellular membranes, travels to nucleus and becomes dispersed in other subcellular locations. It binds to several membrane-resident phospholipids with preference for phosphatidylinositol monophosphates and forms oligomers. Mutations of the cationic amino acids present in the two γ-core motifs of this defensin that eliminate oligomerization also knockout its ability to induce membrane permeabilization and fungal growth arrest. MtDef5 is the first bi-domain plant defensin that exhibits potent broad-spectrum antifungal activity, recruits multiple membrane phospholipids and forms oligomers in their presence. These findings raise the possibility that MtDef5 might be useful as a novel antifungal agent in transgenic crops.

## Introduction

Plants have evolved an innate immune system for defense against pathogenic microorganisms. This ancient defense system provides nonspecific broad-spectrum resistance against microbial invasion. The innate immunity of plants comprises fortification of cell wall, hypersensitive response and production of antimicrobial compounds and antimicrobial peptides (AMPs). AMPs serve as one of the first lines of defense against pathogen invasion and are one of the key contributors to innate immunity in plants^1^.

Defensins represent a major class of AMPs found in almost all eukaryotes. These cysteine-rich peptides are diverse members of one of several AMP families in plants^2^. They are 45–54 amino acids in length and their constitutive, regulated or induced expression confers durable resistance to fungal pathogens^3,4^. In the oxidized form, they contain an invariant tetradisulfide array and share a cysteine-stabilized αβ backbone in which one α-helix is stabilized through disulfide bonding to three antiparallel β strands. Despite their structural similarity, plant defensins vary greatly in their amino acid sequences revealing a rich diversity of variants^5^. This variation in their primary amino acid sequences likely confers diverse physiological functions to members of the plant defensin family^6,7^.

The best known property of the cationic plant defensins is their ability to inhibit the growth of fungal pathogens *in vitro* and *in planta*. However, their mechanisms of action (MOA) are not yet fully understood. It is now recognized that plant defensins vary in their MOA^3,8,9^. Interaction of some plant defensins with specific cell wall/plasma membrane resident sphingolipids results in the induction of cell wall stress, accumulation of ceramides and reactive oxygen species (ROS), and ultimately cell death^2,3^. During the last few years, antifungal plant defensins have been discovered that gain entry into fungal cells and bind to bioactive plasma membrane resident phospholipids^10–14^. The vacuole-localized defensins NaD1 and TPP3 from *Nicotiana alata* and *Solanum lycopersicum*, respectively, bind to plasma membrane localized phosphoinositides, in particular, phosphatidylinositol 4, 5-bisphosphate (PIP_2_), and form PIP_2_-dependent oligomeric complexes^13,15^. *Medicago truncatula* defensin MtDef4 and *N*. *suaveolens* defensin NsD7 target phosphatidic acid (PA) and this interaction is important for their antifungal activity^14,16^. NsD7 forms PA-dependent oligomeric complexes and disrupts the fungal plasma membrane^16^. These findings suggest that plant defensins recruit one or more bioactive phospholipids to oligomerize, induce membrane disruption and trigger fungal cell death.

The genome of the model legume *M*. *truncatula* is predicted to encode 63 defensins with a tetradisulfide array^17^. Here, we report characterization of a novel bi-domain defensin MtDef5 encoded by a single gene in the genome of this plant. This defensin consists of two 50-amino acid defensin domains that are linked by a 7-amino acid peptide. We show that it inhibits the growth of fungal pathogens tested at submicromolar concentrations. It disrupts the plasma membrane of fungal cells and induces accumulation of ROS. MtDef5 also binds to several membrane-resident phospholipids with strong preference for phosphatidylinositol monophosphates (PIP). We provide further evidence that this interaction with phospholipids leads to oligomerization of MtDef5 regardless of its affinity for them. Using site-directed mutagenesis, we demonstrate that the cationic amino acid residues present in each of the two γ-core motifs of this defensin are required for its oligomerization and antifungal activity.

## Results

### MtDef5 is a bi-domain defensin from *Medicago truncatula*

**Figure 1:**
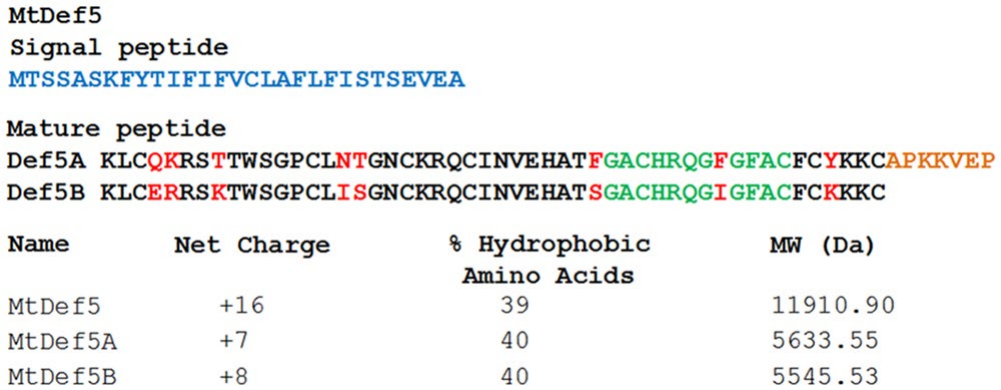
The deduced amino acid sequences of bi-domain MtDef5. The 29-amino acid signal peptide sequence is shown in blue. The mature MtDef5 protein containing its two defensin domains, MtDef5A and MtDef5B, each 50 amino acids in length, is shown in black. MtDef5A and MtDef5B are connected by a 7-amino acid linker shown in orange. Amino acid differences between the two domains are shown in red. The γ-core motif of each domain is shown in green. The net charge, hydrophobicity and molecular weight of the bi-domain MtDef5 and its two domains are shown.

Previously, our search of the *M*. *truncatula* GeneIndex (MtGI 4.0) revealed the expression of a bi-domain defensin gene identified as a Tentative Consensus 87273^18^. The genomic locus *(MTR_8g012775)* representing TC87273 encodes a signal peptide of 29 amino acids and a mature protein of 107 amino acids (Fig. 1). The sequence of the predicted mature protein revealed the presence of two defensin domains, designated MtDef5A and MtDef5B, each 50 amino acids in length, that are connected by a 7-amino acid linker sequence APKKVEP. MtDef5 contains a net charge of +16 and 39% amino acids with hydrophobic side chains. The two domains share 84% amino acid identity (Fig. 1). The tertiary protein structure of MtDef5 predicted by Raptorx web portal (http://raptorx.uchicago.edu/) indicates each domain to fold independently into a cysteine-stabilized alpha-beta (CSαβ) configuration consisting of three antiparallel β strands and one α-helix stabilized by four disulfide bridges. Each domain contains a γ-core motif GACHRQG(F/I)GFAC which connects the two anti-parallel β_2_ and β_3_ strands and carries a net positive charge of +2 and 4 hydrophobic amino acids. This nearly identical motif present in each domain differs in sequence from that of all previously characterized single domain plant defensins.

### MtDef5 exhibits potent broad-spectrum antifungal activity

**Figure 2:**
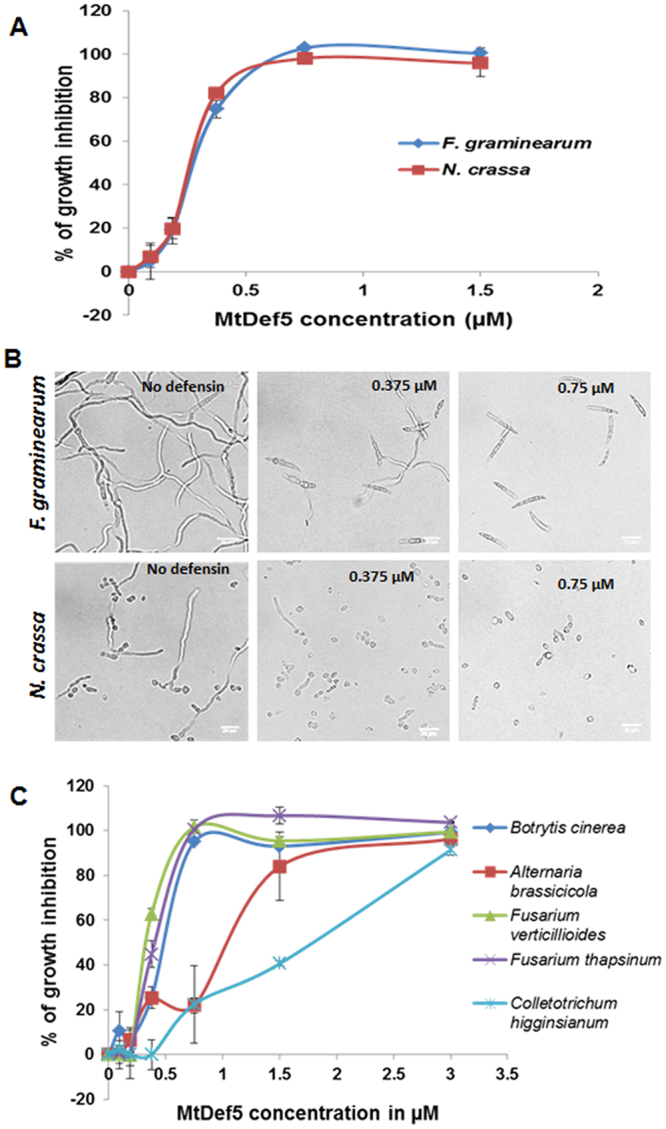
The bi-domain MtDef5 inhibits fungal growth at submicromolar concentrations. (**A**) Quantitative assessment of the inhibition of fungal growth of *F*. *graminearum* and *N*. *crassa* at different concentrations of MtDef5. Values are means of three replications. Error bars indicate standard deviations. (**B**) The growth of both fungi is completely inhibited at a concentration of 0.75 µM. Bar = 20 µm. (**C**) MtDef5 exhibits broad-spectrum *in vitro* antifungal activity against fungal pathogens.

Plant defensins exhibit inhibitory activity against filamentous fungi *in vitro* at micromolar concentrations. Since MtDef5 consists of two domains and is highly cationic, we determined its *in vitro* antifungal activity against several filamentous fungi. MtDef5 expressed in *Pichia pastoris* was determined to have the molecular mass of 12915.35 Da, ~1025 Da higher than the calculated mass of 11887.74 Da (Supplementary Figure S1). The α-factor signal peptide was incorrectly processed adding EEGVSLEKR at the N-terminus of the mature MtDef5 peptide sequence. However, this additional sequence of 9 amino acids has minor effect on the overall net charge and hydrophobicity of MtDef5 (Supplementary Table S1) and exhibits no antifungal activity against *F*. *graminearum* (Figure S2). Therefore, we decided to proceed with testing the antifungal activity of MtDef5 with the observed mass of 12915.35 Da. MtDef5 inhibited the growth of *F*. *graminearum* and *N*. *crassa* with an IC_50_ value of 0.25–0.3 µM and minimal inhibitory concentration (MIC) of 0.70–0.75 µM (Fig. 2A,B). It also inhibited the growth *in vitro* of several other filamentous plant fungal pathogens including *F*. *verticilloides, F*. *thapsinum, Alternaria brassicicola*, *Colletotrichum higginsianum* and *Botrytis cinerea* (Fig. 2C). *C*. *higginsianum* was the least sensitive pathogen among this group of fungal pathogens with an IC_50_ value of 1.75 µM and MIC of 3.0 µM. The antifungal activity of MtDef5 against *F*. *graminearum* was also compared with that of each of its two domains. From the data presented in Supplementary Table S2, it is clear that MtDef5 has more potent antifungal activity than its two domains, MtDef5A and MtDef5B. In addition, the *in vitro* antifungal activity of MtDef5A and MtDef5B, when used in combination, did not equal to that of MtDef5 suggesting the linker peptide APKKVEP contributes to the potent antifungal activity of the bi-domain MtDef5.

### MtDef5 permeabilizes fungal plasma membrane and induces accumulation of ROS

**Figure 3:**
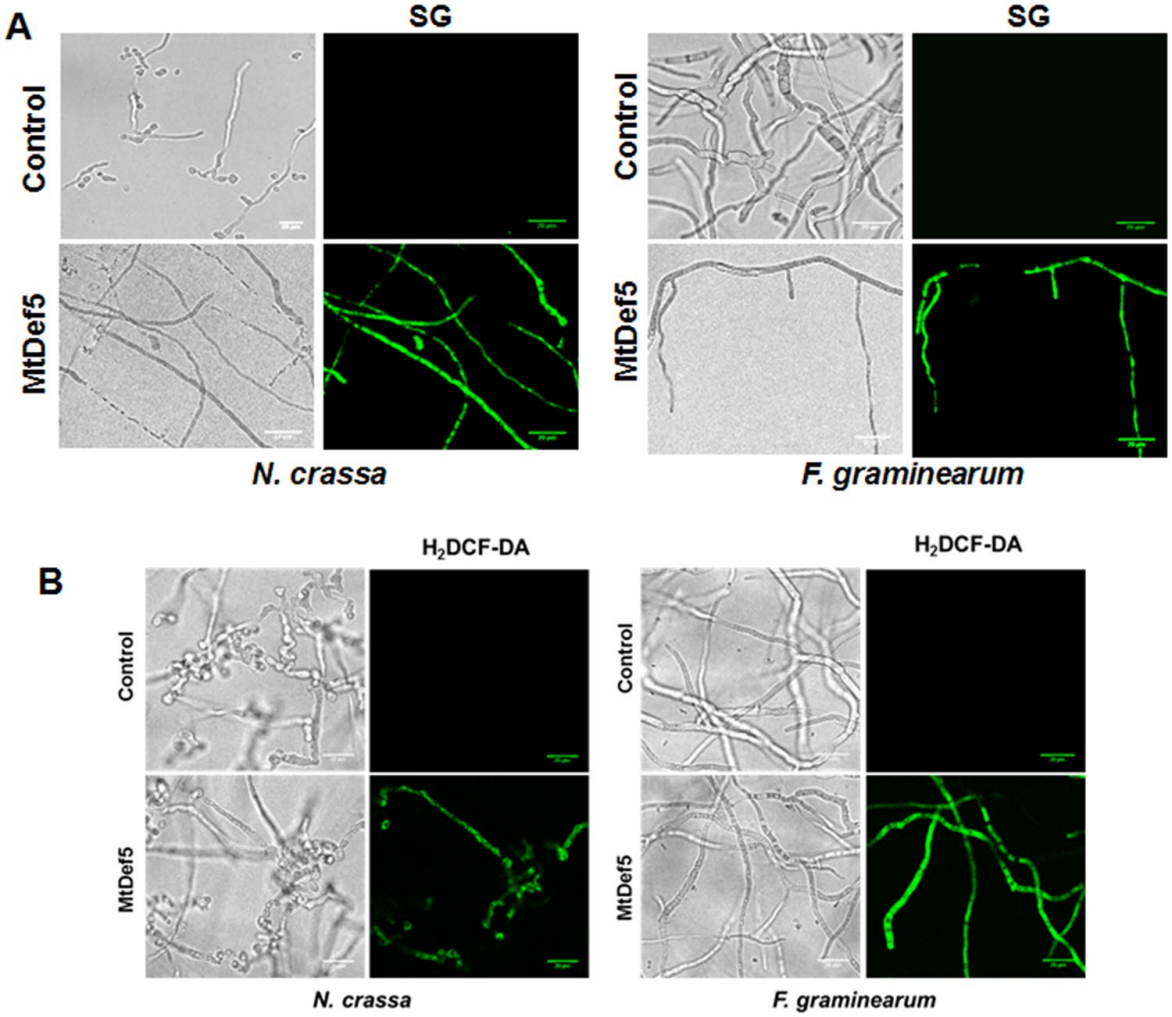
MtDef5 permeabilizes the fungal plasma membrane and induces accumulation of ROS. (**A**) Fluorescence images of SG binding to the nuclei of *N*. *crassa* and *F*. *graminearum*, following treatment with 0.75 µM of MtDef5 + 0.5 µM SG. Bar = 20 µm. (**B**) Confocal microscopy showing ROS production (H_2_DCF-DA, green fluorescence) in *F*. *graminearum* and *N*. *crassa* cells after treatment with 0.75 μM MtDef5 for 60 min. Bars = 20 μm.

Cationic antimicrobial peptides are known to permeabilize the plasma membrane of their target microbes. Thus, plant defensins have been previously shown to permeabilize the plasma membrane of their target fungi^12,19,20^. The SYTOX Green (SG) uptake assay was used to test if MtDef5 permeabilizes the plasma membrane of *F*. *graminearum* and *N*. *crassa*. SG is a dye which is only taken up by cells with a compromised plasma membrane and its fluorescence increases >500-fold upon binding to nucleic acids thus allowing quantitative analysis and fluorescence microscopy. The uptake of SG was assessed using confocal microscopy in fungal hyphae treated with 0.75 µM MtDef5. SG uptake was visible in the hyphal cells of both fungi after 2 h of treatment with MtDef5 (Fig. 3A). This result suggests that membrane disruption likely contributes to the antifungal action of MtDef5.

Antifungal defensins with different MOA are known to converge on a vital cellular hub representing the production of ROS to achieve fungal killing^10,21,22^. Therefore, we examined if the fungal plasma membrane permeabilization induced by MtDef5 is paired with a commensurate rise in the production of ROS in the cells of *F*. *graminearum* and *N*. *crassa*. The production of ROS was monitored by confocal microscopy using the cell permeant ROS indicator 2′,7′-dichlorodihydrofluorescein diacetate (H_2_DCF-DA) following treatment of fungal hyphae with MtDef5. Upon production of ROS, the nonfluorescent H_2_DCF-DA is converted to highly fluorescent 2′,7′-dichlorofluorescein (DCF). Accumulation of intracellular ROS was observed in both *F*. *graminearum* and *N*. *crassa* hyphae following exposure to MtDef5 (Fig. 3B) indicating ROS-mediated apoptosis- or necrosis-like process involved in fungal cell killing by this defensin.

### Internalization and subcellular localization of MtDef5

**Figure 4:**
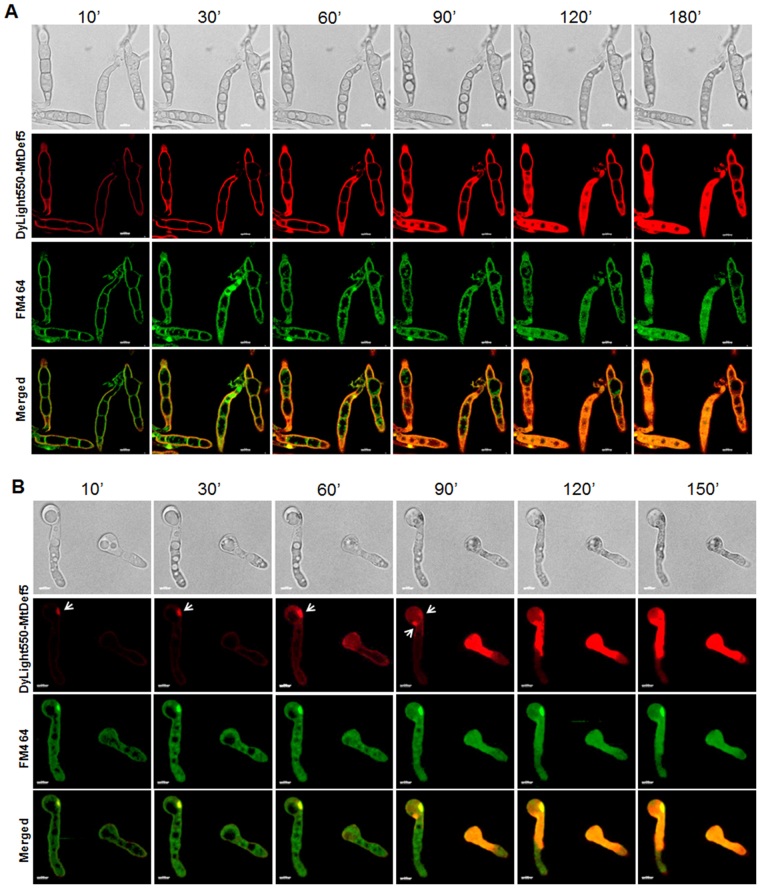
MtDef5 uses different modes of entry in *F*. *graminearum* and *N*. *crassa*. (**A**) Time lapse confocal microscopy of *F*. *graminearum* germlings showing the internalization of DyLight550-MtDef5 and its co-localization with membrane-selective dye FM4-64. Peptide binds to the surface of the cell uniformly (10′) followed by internalization within the cell (60′, 90′). (**B**) In *N*. *crassa* germlings, DyLight550-MtDef5 is internalized through random entry sites on the cell surface as indicated by an arrow. Note DyLight550-MtDef5 recognizes the entry site (10′) and is subsequently internalized into the cell (60′, 90′). Fungal germlings were dual labeled with 1.5 µM DyLight550-MtDef5 and 5 µM FM4-64. Bar = 5 µm.

In order to determine if MtDef5 acts via the extracellular side or gains entry into fungal cells, it was labeled with the fluorophore DyLight550 and its uptake was monitored using the live-cell imaging of conidia and conidial germlings of *F*. *graminearum* and *N*. *crassa*. DyLight550 labeled MtDef5 exhibited reduced antifungal activity with an MIC of 1.5 µM compared with an MIC of 0.75 µM for the unlabeled MtDef5. At MIC, entry of DyLight550 labeled MtDef5 within conidial germlings was monitored by confocal microscopy and time-lapse imaging. In *F*. *graminearum*, within 10 min of treatment, MtDef5 readily bound to the conidial surface uniformly and a considerable increase in binding was evident over the next 60 min. After approximately 60 min, MtDef5 started entering into the growing hyphal tip first and, in 90 to 120 min, it was internalized by most of the fungal cell (Fig. 4A and Supplementary Video S1). In sharp contrast, MtDef5 did not bind to the conidial surface uniformly in *N*. *crassa*. Instead, it entered into the cell by making random entry sites (Fig. 4B and Supplementary Video S2). Within 10 min, MtDef5 bound to specific sites of the cell surface and accumulated there before entering the cell. After ~30 min, MtDef5 started entering the cell from the entry sites. The membrane selective dye FM4-64 is also co-localized at the entry sites indicating perhaps endosomal uptake of the defensin (see below). Accumulation of MtDef5 inside the *N*. *crassa* cells continued to increase over time until it was almost completely internalized by the cells after 2 h of treatment (Fig. 4B). Thus, the uptake process of MtDef5 in *N*. *crassa* is strikingly different from that in *F*. *graminearum*.

**Figure 5:**
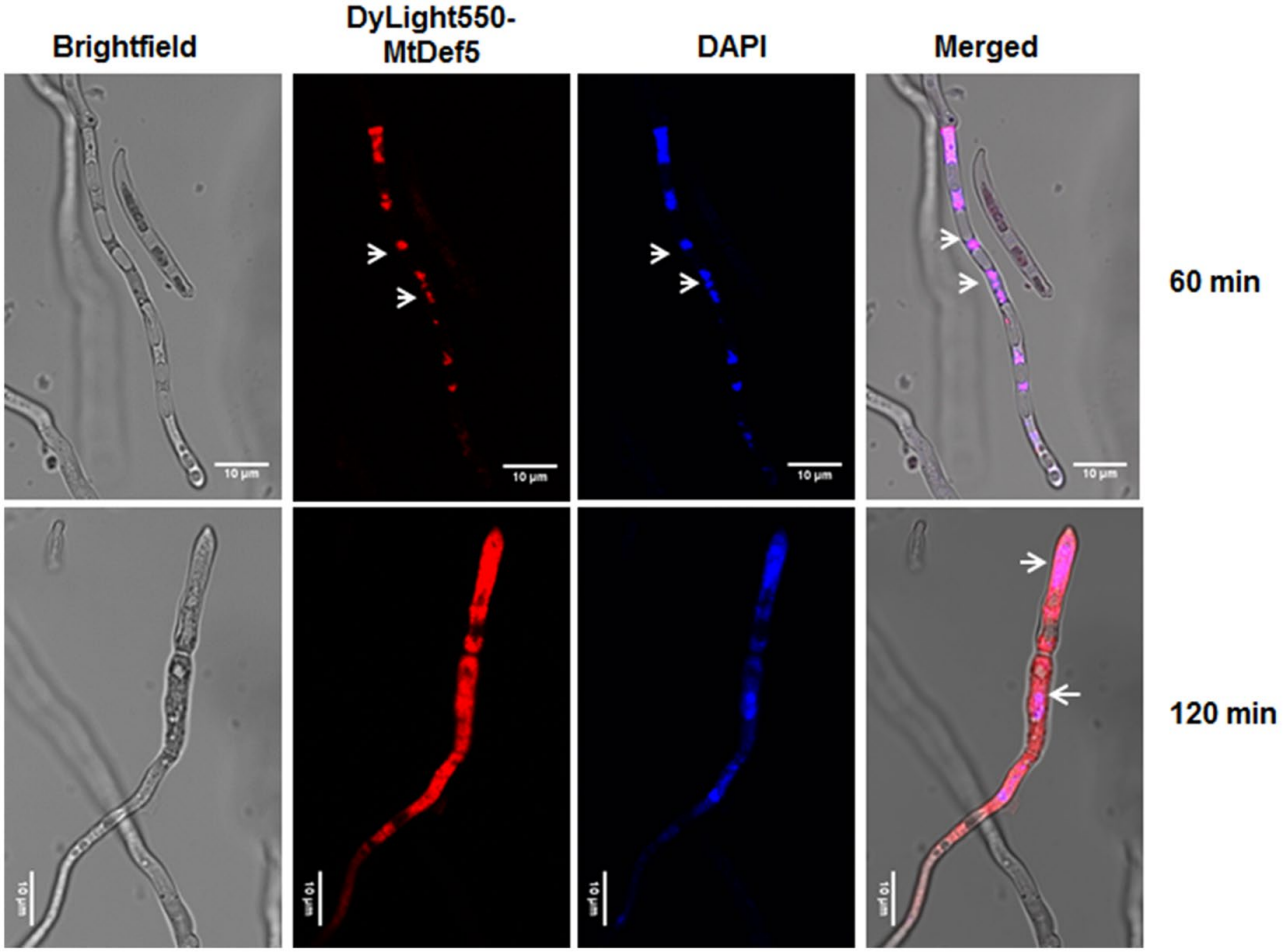
MtDef5 is targeted to the nucleus initially. Dual labeling of the germlings of *F*. *graminearum* with 1.5 µM DyLight550-MtDef5 (red) and nuclear-staining dye DAPI (blue) shows that DyLight550-MtDef5 is localized in the nuclei as indicated by arrows.

Intracellular localization of MtDef5 was further investigated by examining co-localization of DyLight550 labeled MtDef5 with the membrane selective dye FM4-64^23^, membrane permeant dye SG which binds to nucleic acid and DNA specific stain 4′,6-diamidino-2-phenylindole **(**DAPI) in germlings of *F*. *graminearum* and *N*. *crassa*. MtDef5 is uniformly dispersed in the cells of both fungi. Dual labeling with FM4-64 showed that MtDef5 is co-localized with cellular membranes in cells of both fungi (Fig. 4). As shown in Fig. 5, MtDef5 accumulates first in the nucleus of *F*.*graminearum* cells (red) as revealed by its co-localization with a blue fluorescent DNA stain DAPI. The subcellular localization of MtDef5 in the nucleus and subsequent degeneration of this organelle was further confirmed by demonstrating co-localization of DyLight550-labeled defensin with DNA-complexed SG (green) as shown in the Supplementary Fig. S3 and Supplementary Video S3 and S4.Following entry of this defensin into fungal cells, we also observed a gradual increase in the size of the vacuole and ultimately its burst. The cell lysis was also observed within the timeframe of live cell imaging in both fungi (Supplementary Video S3 and S4). We conclude that MtDef5 is targeted to the nucleus, intracellular membranes and other subcellular locations following its entry into fungal cells suggesting the involvement of perhaps multiple targets contributing to fungal cell killing.

### MtDef5 binds to multiple phospholipids with strong preference for PIP

**Figure 6:**
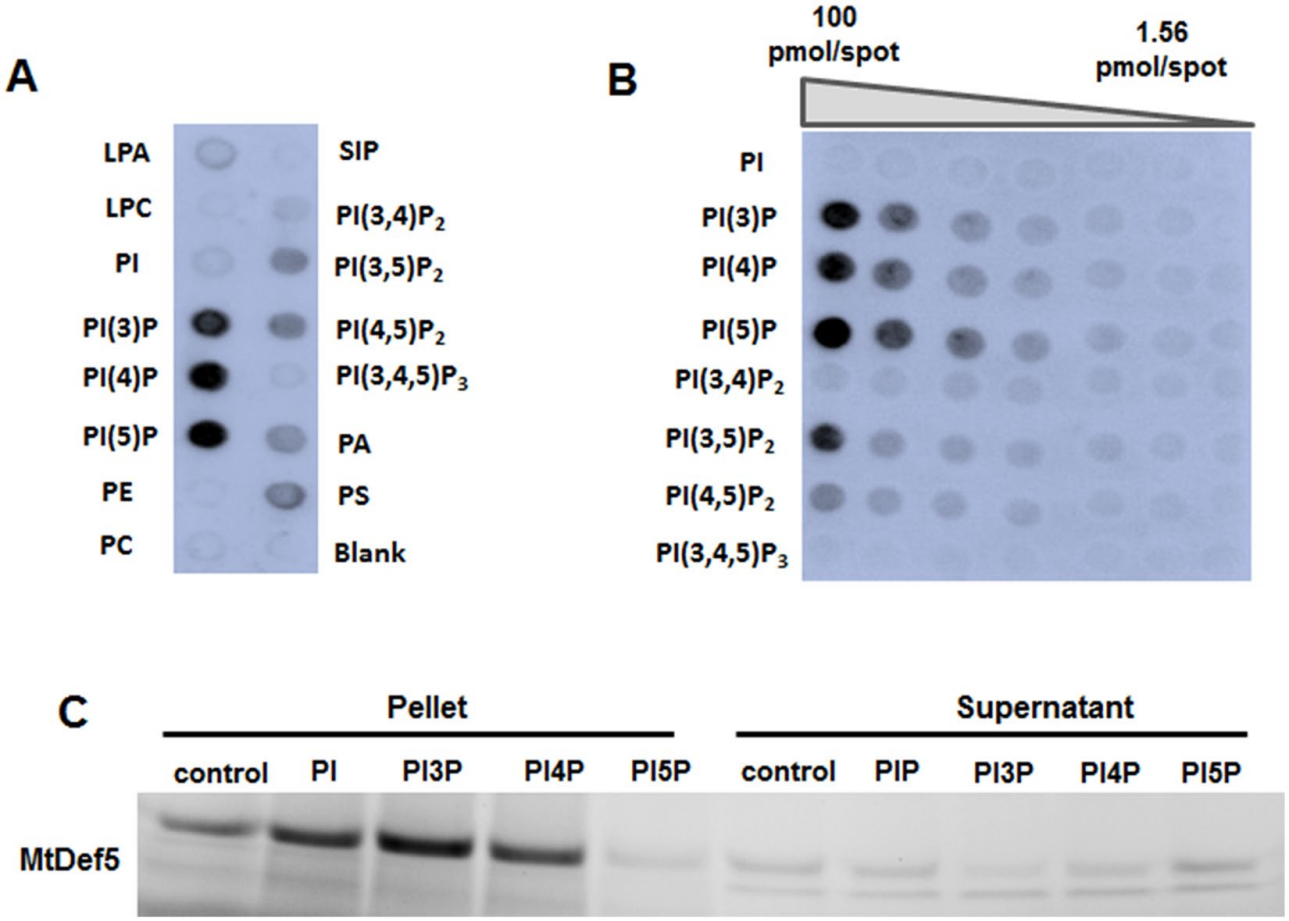
MtDef5 binds to multiple phospholipids but with higher affinity for PIP. (**A**) PIP strip showing the binding of MtDef5 to PI3P, PI4P and PI5P and also to PI4,5P_2_, PI3,5P_2_, PA and PS. (**B**) PIP array displays the relative binding of MtDef5 to phospholipids. (**C**) PolyPIPosome binding assay shows that MtDef5 binds with higher affinity to PI3P and PI4P as compared to PI5P. The full length gel is presented in Supplementary Figure S4.

Plant defensins target membrane phospholipids as part of their *modus operandi*. The characterization of defensin-phospholipid interactions can provide novel information regarding the functions of defensins relevant to their subcellular targeting and their potential effects on fungal signaling and vesicle trafficking. To assess the ability of MtDef5 to bind to membrane phospholipids, a protein-lipid overlay assay was performed with various biologically active phospholipids immobilized on a lipid strip (Echelon Bioscience, UT). We found that MtDef5 strongly binds to PI3P, PI4P and PI5P. However, it also shows somewhat weaker binding to PI3,5P_2_, PI4,5P_2_, PA and phosphatidylserine (PS) (Fig. 6A). Based on this lipid binding profile, we selected a more specific lipid array spotted with PI and PIPs at different concentrations. This experiment again confirmed higher affinity of MtDef5 to the monophosphorylated forms of PI with lower affinity for PIP_2_ and lack of affinity for nonphosphorylated PI. Consistent with the initial results obtained using PIP strips, we observed binding of MtDef5 to all three PIPs even at 12.5 pmoles per spot indicating high affinity of this defensin to these bioactive phospholipids (Fig. 6B).

To further validate the binding of MtDef5 to PIP, a liposome binding assay was performed using the commercially available PIP containing liposomes (Echelon Bioscience, UT). As shown in Fig. 6C, MtDef5 showed a preference for binding to PI3P and PI4P, but not for PI5P. Some MtDef5 was also detected in the pellet of liposomes containing PC/PE or PI, indicating PIP-independent binding (Fig. 6C). In summary, MtDef5 binds promiscuously to multiple membrane phospholipids but has a preference for PI3P and PI4P.

### MtDef5 forms oligomers in presence of PIP, PA and PI

**Figure 7:**
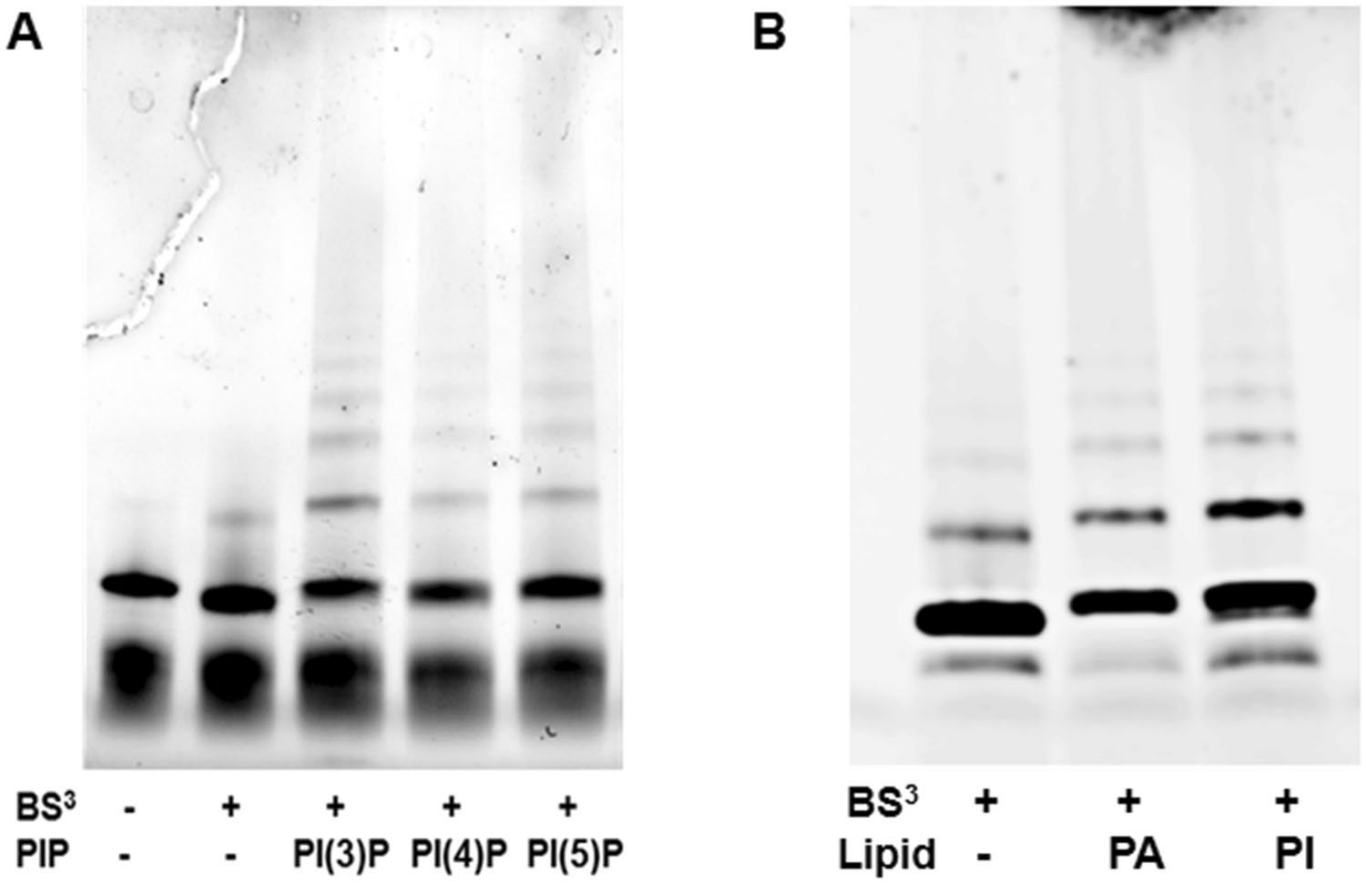
MtDef5 forms oligomers only in presence of PIP. (**A**) MtDef5 forms higher order oligomers in presence of PI3P, PI4P and PI5P as revealed by a protein cross-linking with BS^3^ followed by SDS-PAGE and visualized using a Bio-Rad ChemiDoc XRS + system. (**B**) MtDef5 also forms oligomers in presence of PA and PI. The full length gel is presented in Supplementary Figure S5.

The ability of defensins to self-assemble into oligomers is important for their antimicrobial activity. We tested the ability of MtDef5 to oligomerize by treating MtDef5 with the biochemical cross-linker BS^3^ in absence and presence of PI3P, PI4P, PI5P, PI and PA. Separation of cross-linked protein complexes by a gradient SDS-PAGE indicated that MtDef5 formed covalently linked higher-order oligomers in presence of all three PIP (Fig. 7A). Surprisingly, it also formed oligomers in presence of PI and PA with whom it shows weak binding (Fig. 7B). This ability of MtDef5 to form oligomers in presence of multiple phospholipids is striking and may account for its potent antifungal activity against a broad range of fungi.

### The γ-core motif mediated oligomerization, but not phospholipid binding, is important for the antifungal activity of MtDef5

**Figure 8:**
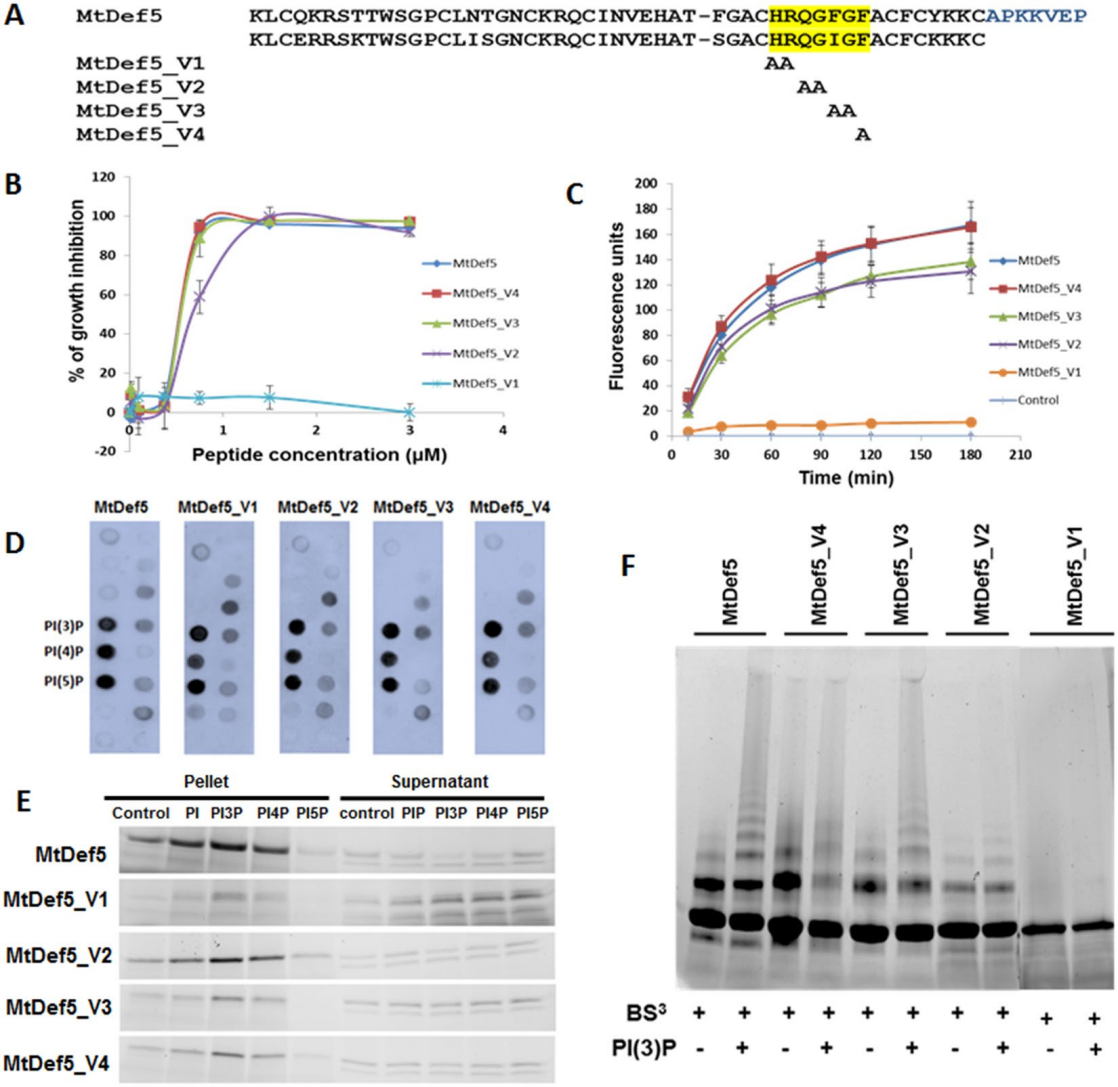
γ-core motif sequences of MtDef5 are critical for membrane permeabilization, oligomerization and antifungal activity of MtDef5, but not for PIP binding. (**A**) Sequence of MtDef5 and its variants. Amino acid residues of the two γ-core motifs (highlighted) were mutated to alanine (**A**). (**B**) Quantitative assessment of the *in vitro* antifungal activity of MtDef5 and its variants against *F*. *graminearum* conidia after 20 h of treatment. Values are means of three replications. Error bars indicate standard deviations. (**C**) Quantitative measurement of fluorescence emitted by hyphae treated with 0.75 µM MtDef5 or its variants plus 0.5 µM of SYTOX Green. Values are means of three replications. Error bars indicate standard deviations. (**D**) Interaction of MtDef5 and it variants to phospholipids. PIP strip shows that MtDef5 and it variants strongly bind to PI3P, PI4P and PI5P and also to PI4,5P_2_, PI3,5P_2_, PA and PS. (**E**) PolyPIPosome binding assays of MtDef5 and its γ-core motif variants. It shows that MtDef5 binds to PI3P and PI4P with higher affinity than PI5P. It also binds to PI. The full length gels are presented in Supplementary Figure S6. (**F**) Oligomerization of MtDef5 γ-core motif variants in presence of PI(3)P. MtDef5_V1 variant containing the H36A, R37A, H93A and R94A substitutions loses its ability to oligomerize in presence of PI3P. The full length gel is presented in Supplementary Figure S7.

The γ-core motif has been shown to be important for phospholipid binding and antifungal activity of plant defensins^14^. A four-membered MtDef5 variant family was designed and prepared to identify amino acid residues mediating phospholipid binding, oligomerization and antifungal activity in the γ-core motif of this defensin. Since the amino acid sequence of this motif is nearly identical in both domains, we used alanine scanning mutagenesis to substitute two adjacent amino acids of each motif simultaneously for alanine generating four variants (Fig. 8A). These variants were each expressed in *P*. *pastoris* and purified by ion exchange chromatography. They were subsequently tested for phospholipid binding, plasma membrane permeabilization, oligomerization and antifungal activity. The antifungal activity of MtDef5 variants was tested against *F*. *graminearum*. As shown in Fig. 8B, MtDef5^F40A, G41A/I97A, G98A^ (MtDef5_V3) and MtDef5^F42A/F99A^ (MtDef5_V4) retained wild-type (WT) antifungal activity. However, MtDef5^H36A, R37A/H93A, R94A^ (MtDef5_V1) almost completely lost its antifungal activity and MtDef5^Q38A, G39A/Q95A, G96A^ (MtDef5_V2) showed 2-fold reduction in its antifungal activity against *F*. *graminearum* relative to that of the WT MtDef5. These variants were also tested for their ability to permeabilize the plasma membrane of *F*. *graminearum* using the SG uptake quantification assay. As shown in Fig. 8C, WT MtDef5 and MtDef5 ^F42A/F99A^ (MtDef5_V4) exhibited faster and higher uptake of SG than the MtDef5 ^Q38A, G39A/Q95A, G96A^ (MtDef5_V2) and MtDef5 ^F40A, G41A/I97A, G98A^ (MtDef5_V3). In contrast, MtDef5 ^H36A, R37A/H93A, R94A^ (MtDef5_V1) lost its ability to permeabilize the fungal plasma membrane almost completely (Fig. 8C).

The ability of each variant to interact with PIPs was examined by performing a protein-lipid overlay assay. Surprisingly, the protein-lipid overlay assay revealed that MtDef5 γ-core motif variants did not significantly alter their lipid binding profiles and retained the WT MtDef5 PIP binding affinity and specificity (Fig. 8D). Some non-specific binding to phospholipids on PIP strips is known to occur by the presence of small amount of misfolded peptide and the non-specific binding can be blocked by adding sub-stoichiometric amounts of DnaK^24^. To eliminate non-specific binding, the WT and variant peptides were each incubated with DnaK and subsequently used on PIP strips. However, no non-specific binding to any of the phospholipids was observed indicating there was little or no misfolded peptide (data not shown) in the WT or γ-core motif variants used in the assay. By contrast, in the liposome binding assay, all γ-core motif variants exhibited somewhat reduced binding to all three PIPs. In particular, MtDef5 ^H36A, R37A/H93A, R94A^ (MtDef5_V1) showed a much weaker binding affinity for all three PIPs as compared to that of the WT MtDef5 and the other three MtDef5 γ-core motif variants (Fig. 8E). Like MtDef5, each γ-core motif variant was also detected in the pellet of liposomes containing PC/PE or PI, indicating PIP-independent binding (Fig. 8E). MtDef5 γ-core motif variants were further investigated for their capacity to form PIP-dependent oligomer formation by protein cross-linking. The oligomer formation by MtDef5_V2 and MtDef5_V3 variants in presence of PIP was similar to that of the WT MtDef5. MtDef5_V4 variant consistently showed less higher-order oligomer formation than the WT MtDef5. However, MtDef5_V1 variant containing the H36A, R37A, H93A and R94A substitutions showed complete loss of oligomer formation in presence of PI3P. It also did not form any oligomers in presence of either PI or PA (data not shown). We conclude that histidine and arginine residues present in the γ-core motif of each domain are critical for oligomerization and antifungal activity of MtDef5; however, the antifungal activity of this defensin does not appear to correlate tightly with its ability to bind phospholipids.

## Discussion

In the present study, we report the presence of a single gene in the genome of *M*. *truncatula* encoding a novel bi-domain antifungal defensin MtDef5 in which two domains, 50 amino acids each, are linked by a 7-amino acid peptide APKKVEP. The genome of *M*. *truncatula* is predicted to encode 63 defensins^17^; however, MtDef5 is the only bi-domain defensin encoded by a single gene in this model legume plant. A homolog of MtDef5 sharing high sequence homology is also present in a crop legume *M*. *sativa*. It is likely that this bi-domain defensin arose through a fusion of two recently duplicated genes encoding single domain defensins in the genomes of these two legumes. The biological function of this defensin in *Medicago* spp. remains to be determined.

Consistent with its high cationicity and hydrophobicity, we find that MtDef5 exhibits potent antifungal activity *in vitro* against several filamentous fungal pathogens at submicromolar concentrations. Its IC_50_ value below 500 nM against several fungi has been rarely reported for other antifungal plant defensins. *N*. *alata* defensin NaD1 inhibits the growth of filamentous fungi with an IC_50_ value ranging from 0.75 to 1.0 µM^10^. An IC_50_ value of 80 nM was reported for antifungal activity of RsAFP2 against *Pyricularia oryzae* ^25^ and 100 nM for HsAFP1 and AhAMP1 against *Septoria tritici* and *Leptosphaeria maculans* ^26^, respectively. It should be noted here that MIC values of different defensins depend upon the ionic strength of the fungal growth media used. Based on its MIC value of 750 nM for antifungal activity against *F*. *graminearum*, MtDef5 is 8- and 32-fold more potent than MtDef4 and MsDef1, respectively, when assayed for antifungal activity under the same conditions^27^. Thus, MtDef5 is one of the most potent broad-spectrum antifungal plant defensins expressed in *M*. *truncatula*.

In the present study, we have conducted several experiments that shed light on the mechanisms governing the highly potent antifungal activity of MtDef5. Plant defensins exert their broad-spectrum antifungal activity using multiple mechanisms including cell membrane permeabilization^13–15,20,28^. At MIC, MtDef5 rapidly permeabilizes the plasma membrane of *F*. *graminearum* and *N*. *crassa* as shown by the uptake of SG dye within minutes. In sharp contrast, MtDef4 permeabilizes the plasma membrane of *F*. *graminearum*, but not of *N*. *crassa* ^29^. It is likely that, the antifungal action of MtDef5, unlike that of MtDef4, involves plasma membrane permeabilization of all its target fungi. MtDef5 challenge also induces accumulation of ROS in fungal cells. ROS has been reported to cause membrane damage and loss of organelle integrity. Its accumulation has been also reported as part of the antifungal action of plant defensins NaD1^10^ and RsAFP2^30^. We propose that plant defensins including MtDef5 that exhibit different MOA converge on ROS accumulation as a central hub for initiating cell death in their target fungi.

Plant defensins, NaD1, TPP2, Psd1 and MtDef4, are internalized and accumulate intracellularly in their target fungi^10–14^. The confocal microscopy of *F*. *graminearum* and *N*. *crassa* cells treated with the fluorescently labeled MtDef5 has revealed that this defensin is also internalized into the cells of both fungi. Interestingly, we have observed key spatial differences in the entry routes of this defensin in these fungi. In *N*. *crassa*, MtDef5 induces one or more entry points on the cell surface where membrane dye FM4-64 is also co-localized. Thus, the formation of membrane-rich spatially restricted sites on cell surface causes initial injury to *N*. *crassa* and offers a mechanism for its internalization into the cytoplasm. By contrast, in *F*. *graminearum*, MtDef5 binds uniformly to the conidial cell wall before entering its cells. Thus, mechanism for entry of MtDef5 in *N*. *crassa* is different from that in *F*. *graminearum*. As shown previously for *Pisum sativum* defensin 1^11^, MtDef5 also travels first to the nucleus and then becomes dispersed in the cytosol. Rapid expansion of the vacuole is observed in both *F*. *graminearum* and *N*. *crassa* culminating in its burst just before cell lysis. It is worth noting that MtDef5 and MtDef4 use different mechanisms for gaining entry into conidial cells of *N*. *crassa* as observed by live cell imaging. The mechanism of entry of MtDef5 as observed by live cell imaging of *N*. *crassa* is also very different from that of MtDef4^29^. To what extent does translocation of this defensin into fungal cells mediates cell death remains to be determined. However, its MOA likely involves interference with metabolic processes, such as protein synthesis or DNA replication. It could also interact directly with specific membrane components.

The molecular mechanism by which MtDef5 permeabilizes the plasma membrane of fungal cells is unknown. It has been previously reported that direct interaction with plasma membrane resident phospholipids and subsequent formation of oligomeric complexes play a major role in mediating membrane permeabilization by plant defensins, NaD1, TPP3 and NsD7^13,15,16^. The phospholipid binding assays used in our study have revealed that MtDef5 binds with high affinity to PI3P and PI4P indicating that phosphorylation on the third and fourth positions on the inositol ring are critical for lipid head group binding. Some binding to PI5P, PIP_2_, PI, PA and PS is also evident. MtDef5 is the first plant defensin that targets preferentially PIP. Based on the protein-lipid overlay assays, the phospholipid binding profiles of the single domain MtDef5A and MtDef5B are similar to that of the bi-domain MtDef5 (data not shown). However, determination of the quantitative differences in the binding specificity of MtDef5A and MtDef5B to specific phospholipids will require further studies. Thus, the phospholipid binding profile of MtDef5 is different from those of MtDef4 and NsD7 which bind to PA, and also different from those of NaD1and TPP3 which bind preferentially to PIP_2_. Plant defensins evidently utilize a broad “phospholipid code” to identify and attack fungal membranes as part of the first line of defense^16^.

PIP is present in tiny amounts in all eukaryotic cells. It is required for endocytic processes such as protein sorting and membrane trafficking^31^. In filamentous fungi, several phospholipids are found including PA, PI3P, PI4P, PI4,5P_2_ and PI3,5P_2_. Fungal hyphae are highly polarized cells and cellular morphogenesis is driven through PIP_2_-dependent organization at the plasma membrane of the growing tip of hypha^32^. As substrates for synthesis of PI4,5P_2_ and PI3,5P_2_, PIP contributes to the polar tip growth in filamentous fungi. Different roles for PIP binding by MtDef5 can be proposed: that binding of PIP present on the inner leaflet of the plasma membrane leads to oligomerization of MtDef5 and subsequent disruption of plasma membrane and that intracellular PIP may enhance the stability and antifungal activity of MtDef5 upon entry into fungal cells.

It is becoming increasingly clear that oligomerization is a critical mechanistic step underlying the antimicrobial action of defensins. The oligomerization of plant defensins, NaD1, TPP3 and NsD7, is mediated by their interaction with specific phospholipids^13,15,16^. Like NaD1, TPP3 and NsD7, MtDef5 also forms oligomers in presence of PI3P, PI4P and PI5P. Surprisingly, MtDef5 also forms oligomers in presence of PA and PI to which it shows very weak but detectable binding in lipid-protein interaction assays. This ability of MtDef5 to form oligomers by recognizing a broad range of phospholipids is indeed intriguing and may explain its potent broad-spectrum antifungal activity at submicromolar concentrations. NaD1 and TPP3 have been reported to oligomerize in presence of PIP_2_^13,15^, whereas NsD7 has been reported to oligomerize in presence of PA^16^. Recently, both NaD1 and NsD7 have also been reported to oligomerize in presence of PA and PIP_2_^33^. Our findings raise important questions regarding the mechanism of phospholipid-dependent oligomerization of MtDef5. It seems likely that interaction of MtDef5 with a negatively charged phospho group is perhaps sufficient to initiate the oligomerization process. MtDef5 forms nanonet-like structures in presence of PI3P (data not shown). Human α-defensin HD6 and more recently human β-defensin 1 has been reported to form self-assembled nanonets that trap bacteria and restrict their motility^34,35^. However, the biological significance of the PIP-dependent nanonet formation by MtDef5 remains to be determined. As reported earlier for NaD1, TPP3 and NsD7^13,15,16^, the X-ray crystal structural analysis of the MtDef5-PIP complex will help understand the assembly process and molecular structure of nanonets. In particular, it will reveal if MtDef5 binds to PIP in a highly cooperative manner to facilitate oligomerization as reported earlier for NaD1- and TPP3-PIP_2_ complex formation^13,15^.

A major issue surrounding the functional consequences of PIP recognition and oligomer formation by antifungal plant defensins remains unresolved. To test the requirement of PIP binding and oligomerization for membrane permeabilization and antifungal activity, a 2-point site-directed mutagenesis of the γ-core motif [GACHRQG(F/I)GFAC] present in each domain of MtDef5 has been carried out. The cationic amino acids H36 and R37 in MtDef5A and H93 and R94 in MtDef5B are critical for oligomerization of MtDef5. Mutations of these amino acids to alanine completely abolished the ability of MtDef5 to induce membrane permeabilization and inhibit fungal growth. Amino acids Q38, G39, F40, G41, F42, A43 in the γ-core motif of MtDef5A and Q95, G96, I97, G98, F99, A100 in γ-core motif of MtDef5B apparently play a more minor role in mediating oligomerization of MtDef5 and consequently have relatively small but significant effect on its antifungal activity. In future, it will be important to directly identify all PIP-binding residues by performing nuclear magnetic resonance titration of MtDef5 with PIP head group or by structural analysis of the MtDef5-PIP complex using X-ray crystallography. The site-directed mutagenesis of these residues should provide further insight into mechanisms underlying the antifungal activity of MtDef5.

Based on the data presented here, we propose a multistep mechanism for antifungal action of MtDef5. This involves (1) initial interaction of MtDef5 with the fungal cell envelope, (2) binding to plasma membrane resident phospholipids, oligomerization and membrane disruption, (3) internalization into cells, induction of ROS and interaction with as yet unknown intracellular targets, and (4) cell killing. It remains to be determined if all steps are essential for antifungal activity of this defensin. Better understanding of each individual step will help elucidate its MOA in detail.

## Methods

### Growth of Fungal Cultures

*F*. *graminearum* PH-1 was routinely cultured on complete medium (CM)^36^. For production of conidia, the fungus was inoculated into carboxymethyl cellulose medium (CMC) and cultured for 2–5 days at 28 °C with shaking at 180 rpm. *N*. *crassa* was routinely cultured on Vogel’s agar media. *Fusarium verticilloides, Fusarium thapsinum, Botrytis cinerea, Alternaria brassicicola* and *Colletotrichum higginsianum* were cultured on potato dextrose agar.

### Expression and Purification of MtDef5 and Its Variants

The chemically synthesized MtDef5A with four disulfide bonds was obtained from JPT Peptide Technologies (Berlin, Germany). MtDef5 and MtDef5B were generated by recombinant expression in *Pichia pastoris*. The codon-optimized synthetic genes encoding MtDef5 and MtDef5B were obtained from GenScript Corporation (Piscataway, NJ) and subsequently cloned into pPICZ*α*A, as described below. MtDef5 and MtDef5B genes were cloned into the EcoRV site of pUC57. A sequence encoding the XhoI restriction endonuclease and the KEX2 protease site (CTCGAGAAAAGA) was added upstream of the mature coding sequence of each defensin. Two stop codons, along with an XbaI restriction enzyme site (TAGTAATCTAGA) were added downstream of the mature coding sequence. Synthetic genes encoding the MtDef5 γ-core motif variants were custom synthesized by GenScript (Piscataway, NJ). The synthetic genes encoding MtDef5^H36A,R37A/H93A,R94A^ (MtDef5_V1), MtDef5^Q38A, G39A/Q95A, G96A^ (MtDef5_V2), MtDef5^F40A,G41A/I97A,G98A^ (MtDef5_V3) and MtDef5^F42A/F99A^ (MtDef5_V4) were thus generated.

For expression in *Pichia pastoris*, the codon-optimized DNA sequences encoding the mature defensin sequence of MtDef5 and its variants and MtDef5B were cloned between the *Xho*I and *Xba*I sites of pPICZ*α*A vector in frame with the *α*-factor secretion signal sequence without the Glu-Ala repeats at the Kex2 signal cleavage site. The resulting recombinant plasmid containing each defensin sequence was then linearized by digestion with *SacI* restriction enzyme and transformed into *P*. *pastoris* × 33 by electroporation. Transformants were initially selected on YPD (10 g/L yeast extract, 20 g/L peptone, 20 g/L glucose and 20 g/L agar) plates containing 150 µg/mL of zeocin, followed by restreaking the transformants on YPD plates containing 500 µg/mL of zeocin. Finally, transformants that survived at higher zeocin concentration were used for production of MtDef5 protein and its variants. The *P*. *pastoris* transformants were grown in liquid medium containing methanol for induction of each defensin. MtDef5, its variants and MtDef5B secreted into the medium were purified using the CM-Sephadex C-25 cation-exchange chromatography as described previously^27^. Fractions containing defensin were dialyzed against 50 mM Tris, pH 7.6 and subsequently lyophilized. Each lyophilized defensin was re-suspended in nuclease-free water and the protein concentration was determined by NanoDrop spectrophotometry. Purity and size of each defensin were assessed by electrophoresis on a 15% SDS-PAGE gel. From one liter of *P*. *pastoris* culture expressing each defensin, yield of approximately 3 mg of purified protein was obtained. Mass Spectrophotometry analysis of each protein sample was performed by the Proteomics and Mass Spectrophotometry Facility at the Danforth Center.

### Antifungal Assays

*In vitro* antifungal assays were performed using a synthetic fungal medium (SFM) without calcium as described previously^37^. Antifungal activity of MtDef5 and its variants, MtDef5A and MtDef5B was observed by taking bright-field images using the transmitted light channel in a Leica SP8-X confocal microscope. The quantitative fungal growth inhibition was estimated by measuring the absorbance at 595 nm using a Tecan Infinite M200 Pro (Tecan Systems Inc., San Jose, CA) microplate reader at different time points. Statistical data analysis was performed using Microsoft Excel (2010).

### SYTOX Green (SG) Uptake Assay

SG uptake assays were conducted as described^20^. *N*. *crassa* and *F*. *graminearum* conidia (50 µL of 5 × 10^4^ / mL) were allowed to germinate overnight at room temperature in Vogel’s liquid and 2X SFM media, respectively, in a 10 mm microwell of 35 mm glass bottom microwell dish with a No. 1.5 cover glass. Wet filter papers were placed in the container to prevent drying of the conidial suspension. After 16 hours, MtDef5 (50 µL, at concentrations of 0.375 µM and 0.75 µM) and 1 µL of 0.5 µM SG (Thermo-Fisher Scientific, NY) were added to fungal hyphae. Samples were incubated with gentle agitation for 2 hours in dark and mounted on a microscope for imaging. SG was excited at 488 nm and detected at 510–530 nm. A Leica SP8-X confocal microscope was used for all confocal imaging. Control plates with SG, and without MtDef5, were used as negative controls. SG uptake quantification assays of MtDef5 and its variants were performed as described previously^20^. Statistical data analysis was done using Microsoft Excel (2010).

### Intracellular Detection of ROS

Intracellular ROS were detected on 16 h-old germinating conidia after exposure to 0.75 µM MtDef5 for 2 h. After treatment, the incubation mixtures were mixed with 2′,7′-dichlorodihydrofluorescein diacetate (H_2_DCF-DA, Invitogen) at 10 μM final concentration and observations were performed under a confocal microscope (Leica SP8) with the 480 and 527 nm excitation and emission wavelengths.

### Protein-Lipid Interactions

To test the binding properties of MtDef5 and its γ-core motif variants, a protein-lipid overlay experiment was performed using PIP Strips^TM^ (2 × 6 cm nitrocellulose membranes) that are spotted with 100 pmol of various biologically active lipids, respectively (Echelon Biosciences, Salt Lake City, UT). To determine relative degree of the binding of MtDef5, PIP Array (P-6001, Echelon Biosciences, UT) pre-spotted with the concentration gradient of various phosphoinositides was used. The MtDef5-phospholipid interactions were detected using the affinity-purified MtDef5-derived peptide (CQKRSTTWSGP) polyclonal antibody (GenScript, Piscataway, NJ) and HRP-conjugated Goat Anti-Rabbit IgG secondary antibody following the manufacturer’s protocol with minor modifications as described previously^14^. The binding of the peptide polyclonal antibody to MtDef5 was confirmed by Western blot analysis (Supplemental Figure S7). The specificity of the binding of polyclonal antibody used here to MtDef5 was confirmed by Western blot analysis (Figure S8).

### PolyPIPosome Binding Assay

Preferential binding of the MtDef5 and its γ-core motif variants to PIP was further validated by a liposome binding assay. PolyPIPosome binding assays were performed in 200 µL of binding buffer (20 mM HEPES pH 7.4, 120 mM NaCl, 1 mM EGTA, 1 mM MgCl_2_, 1 mg/mL BSA, 0.2 mM CaCl_2_, 5 mM KCl) containing 10 µL each of control PolyPIPosome, PI PolyPIPosome, PI3P PolyPIPosome, PI4P PolyPIPosomes and PI5P PolyPIPosome (Echelon Biosciences). Subsequently, a total of 1.25 µg each of MtDef5 and its γ-core motif variants was added and incubated for 1 h at room temperature. Liposomes were collected by centrifugation at 16, 000 × g for 20 min, and supernatant saved and the pellet was washed three times in 1 mL of binding buffer. Following three washes, pellet was resuspended in 40 µL of 2x Laemmli sample buffer and an aliquot of 20 µL of the supernatant was mixed with 20 µL of sample buffer. Both samples were reduced with 100 mM dithiothreitol (DTT) and separated on a 4–20% (Bio-Rad, TGX Stain-Free™ precast gels) SDS-PAGE gel. The proteins were visualized using a Bio-Rad ChemiDoc XRS + system. Stain-free gels contain a special Trihalo compound, which, when activated, reacts with tryptophan residues in the protein sample to emit a fluorescence signal. For SDS-PAGE, Precision Plus Dual Xtra Protein Standards (2–250 kD) from Bio-Rad (Hercules, CA) were used.

### Defensin Oligomerization Assays

To determine the oligomeric status of MtDef5 and its γ-core motif variants, protein cross-linking experiments were performed in the presence or absence of PIP, PA and PI cross-linked with bis[sulfosuccinimidyl] suberate (BS^3^) substrate^15^. Briefly, MtDef5 and its γ-core motif variants, prepared in 1X PBS buffer, at 1.5 mg/ml (5 µL) were incubated with 2.73 mM PI3P, PI4P or PI5P (1.5 µL) at room temperature for 30 min. The cross-linking reaction was initiated by addition of freshly dissolved water soluble 12.5 mM BS^3^ (0.5 μL) and incubated for 30 min at room temperature. After crosslinking, samples were reduced with 100 mM dithiothreitol (DTT) and separated on a 4–20% (Bio-Rad, TGX Stain-Free™ precast gels) SDS-PAGE. The oligomerization pattern was visualized using a Bio-Rad ChemiDoc XRS + system. For SDS-PAGE, Precision Plus Dual Xtra Protein Standards (2–250 kD) from Bio-Rad (Hercules, CA) were used.

### Live Cell Imaging

MtDef5 defensin was labeled with DyLight550 amine-reactive dye following the protocol provided by the manufacturer (Thermo Scientific, USA). Time-lapse confocal laser scanning microscopy was performed to monitor internalization and subcellular localizations of fluorescently labeled MtDef5 into *N*. *crassa* and *F*. *graminearum* cells. For time lapse imaging, *N*. *cras*sa and *F*. *graminearum* conidia (50 µL of 10^5^/mL) and germlings (4 h old) were placed in 10 mm microwell of 35 mm glass bottom microwell dishes (MatTek Corporation, Ashland, MA). Fungal conidia or germlings were treated with Dylight550-MtDef5 (50 µL of 3 µM) and labeled with either nucleic acid selective dye SG (final concentration: 0.5 µM) or membrane selective dye FM4-64 (final concentration: 5 µM) before mounting on the microscope for time-lapse imaging. Wet filter papers were placed in petri dishes to prevent drying of the conidial suspension. A Leica SP8-X confocal microscope was used for confocal imaging at room temperature in a dark room. DyLight550-MtDef5 was excited at 550 nm and fluorescence detected at 560–600 nm, SG was excited at 488 nm and detected at 510–530 nm, and FM4–64 dye was excited at 550 nm and fluorescence detected at 690–800 nm. Bright-field images were also taken with a transmitted light detector simultaneously. The laser intensity and laser exposure of the cells was kept to a minimum to reduce photobleaching and fungal cell damage. Images were analyzed using ImageJ and Bitplane Imaris softwares.

### DAPI staining of MtDef5-treated *F*. *graminearum*

*F*. *graminearum* conidia germlings (50 µL of 10^5^/mL) were incubated with Dylight550-MtDef5 (50 µL of 3 µM) for 1 h or 2 h at room temperature in 10 mm microwell of 35 mm glass bottom microwell dishes (MatTek Corporation, Ashland, MA). Fungal germlings were fixed in 4% paraformaldehyde and washed twice with PBS. DAPI stain solution was prepared by diluting 300 µM DAPI intermediate in PBS (dilution 1:1,000) to make final concentration 300 nM. Nuclear staining was performed by the addition of 100 µL of 300 nM DAPI for 15 min followed by three washes with PBS and culture dish was mounted immediately on the confocal microscope for imaging. DyLight550-MtDef5 was excited at 550 nm and fluorescence was detected at 560–600 nm and DAPI was excited at 405 nm and fluorescence was detected at 430–461 nm.

### Data Availability

All data generated or analyzed during this study are included in this published article

(Supplementary Information files).

## Electronic supplementary material

Supplementary Information

Supplementary Video S1

Supplementary Video S2

Supplementary Video S3

Supplementary Video S4

## References

[1] Goyal RK, Mattoo AK (2014). Multitasking antimicrobial peptides in plant development and host defense against biotic/abiotic stress. Plant Sci.

[2] van der Weerden NL, Bleackley MR, Anderson MA (2013). Properties and mechanisms of action of naturally occurring antifungal peptides. Cell Mol Life Sci.

[3] De Coninck B, Cammue BPA, Thevissen K (2013). Modes of antifungal action and in planta functions of plant defensins and defensin-like peptides. Fungal Biology Reviews.

[4] Kaur J, Sagaram US, Shah D (2011). Can plant defensins be used to engineer durable commercially useful fungal resistance in crop plants?. Fungal Biology Reviews.

[5] van der Weerden NL, Anderson MA (2013). Plant defensins: Common fold, multiple functions. Fungal Biology Reviews.

[6] Carvalho AdO, Gomes VM (2009). Plant defensins—Prospects for the biological functions and biotechnological properties. Peptides.

[7] Stotz HU, Thomson JG, Wang Y (2009). Plant defensins: Defense, development and application. Plant Signaling & Behavior.

[8] Sagaram, U. S., Kaur, J. & Shah, D. In *Small Wonders: Peptides for Disease Control* Vol. 1095 *ACS Symposium Series* Ch. 15, 317–336 (American Chemical Society, 2012).

[9] Vriens K, Cammue B, Thevissen K (2014). Antifungal plant defensins: Mechanisms of action and production. Molecules.

[10] van der Weerden NL, Lay FT, Anderson MA (2008). The plant defensin, NaD1, enters the cytoplasm of *Fusarium oxysporum* hyphae. Journal of Biological Chemistry.

[11] Lobo DS (2007). Antifungal *Pisum sativum* Defensin 1 interacts with *Neurospora crassa* cyclin F related to the cell cycle. Biochemistry.

[12] van der Weerden NL, Hancock REW, Anderson MA (2010). Permeabilization of fungal hyphae by the plant defensin NaD1 othrough a cell wall-dependent process. Journal of Biological Chemistry.

[13] Baxter AA (2015). The tomato defensin TPP3 binds phosphatidylinositol (4,5)-bisphosphate via a conserved dimeric cationic grip conformation to mediate cell lysis. Molecular and Cellular Biology.

[14] Sagaram US (2013). Structural and functional studies of a phosphatidic acid-binding antifungal plant defensin MtDef4: Identification of an RGFRRR motif governing fungal cell entry. PLOS ONE.

[15] Poon IKH (2014). Phosphoinositide-mediated oligomerization of a defensin induces cell lysis. eLife.

[16] Kvansakul M (2016). Binding of phosphatidic acid by NsD7 mediates the formation of helical defensin–lipid oligomeric assemblies and membrane permeabilization. Proceedings of the National Academy of Sciences.

[17] Maróti G, Downie JA, Kondorosi É (2015). Plant cysteine-rich peptides that inhibit pathogen growth and control rhizobial differentiation in legume nodules. Current Opinion in Plant Biology.

[18] Hanks JN (2005). Defensin gene family in *Medicago truncatula*: structure, expression and induction by signal molecules. Plant Molecular Biology.

[19] Thevissen K, Terras FRG, Broekaert WF (1999). Permeabilization of fungal membranes by plant defensins inhibits fungal growth. Applied and Environmental Microbiology.

[20] Sagaram US, Pandurangi R, Kaur J, Smith TJ, Shah DM (2011). Structure-Activity determinants in antifungal plant defensins MsDef1 and MtDef4 with different modes of action against *Fusarium graminearum*. PLOS ONE.

[21] Mello EO (2011). Antifungal activity of PvD1 defensin involves plasma membrane permeabilization, inhibition of medium acidification, and induction of ROS in fungal cells. Current Microbiology.

[22] Hayes BME (2013). Identification and mechanism of action of the plant defensin NaD1 as a new member of the antifungal drug arsenal against *Candida albicans*. Antimicrobial Agents and Chemotherapy.

[23] Hickey, P. C., Swift, S. R., Roca, M. G. & Read, N. D. In *Methods in Microbiology* Vol. Volume 34, 63–87 (Academic Press, 2004).

[24] Boddey JA (2016). Export of malaria proteins requires co-translational processing of the PEXEL motif independent of phosphatidylinositol-3-phosphate binding. Nat Commun.

[25] Terras FR (1992). Analysis of two novel classes of plant antifungal proteins from radish (*Raphanus sativus* L.) seeds. Journal of Biological Chemistry.

[26] Osborn RW (1995). Isolation and characterisation of plant defensins from seeds of Asteraceae, Fabaceae, Hippocastanaceae and Saxifragaceae. FEBS Letters.

[27] Ramamoorthy V, Zhao X, Snyder AK, Xu JR, Shah DM (2007). Two mitogen-activated protein kinase signalling cascades mediate basal resistance to antifungal plant defensins in *Fusarium graminearum*. Cell Microbiol.

[28] Bleackley, M. R. *et al*. *Nicotiana alata* defensin chimeras reveal differences in the mechanism of fungal and tumour cell killing and an enhanced antifungal variant. *Antimicrobial Agents and Chemotherapy*, 10.1128/aac.01479-16 (2016). 10.1128/AAC.01479-16 PMC5038239 27503651

[29] El-Mounadi K, Islam KT, Hernández-Ortiz P, Read ND, Shah DM (2016). Antifungal mechanisms of a plant defensin MtDef4 are not conserved between the ascomycete fungi *Neurospora crassa* and *Fusarium graminearum*. Molecular Microbiology.

[30] Aerts AM, François IEJA, Cammue BPA, Thevissen K (2008). The mode of antifungal action of plant, insect and human defensins. Cellular and Molecular Life Sciences.

[31] Balla T (2013). Phosphoinositides: tiny lipids with giant impact on cell regulation. Physiol Rev.

[32] Mähs A (2012). The essential phosphoinositide kinase MSS-4 is required for polar hyphal morphogenesis, localizing to sites of growth and cell fusion in *Neurospora crassa*. PLOS ONE.

[33] Jarva M, Lay FT, Hulett MD, Kvansakul M (2017). Structure of the defensin NsD7 in complex with PIP2 reveals that defensin: lipid oligomer topologies are dependent on lipid type. FEBS Lett.

[34] Chu H (2012). Human alpha-defensin 6 promotes mucosal innate immunity through self-assembled peptide nanonets. Science.

[35] Raschig J (2017). Ubiquitously expressed human beta defensin 1 (hBD1) forms bacteria-entrapping nets in a redox dependent mode of action. PLoS Pathog.

[36] Leslie, J. F. & Summerell, B. A. *The Fusarium laboratory manual*. (Blackwell Publishing, 2006).

[37] Broekaert WF, Terras FRG, Cammue BPA, Vanderleyden J (1990). An automated quantitative assay for fungal growth inhibition. FEMS Microbiology Letters.

