# Supplementary material for: A novel bi-domain plant defensin MtDef5 with potent broad-spectrum antifungal activity binds to multiple phospholipids and forms oligomers
Source: Sci Rep. 2017 Nov 23;7:16157. doi: 10.1038/s41598-017-16508-w (PMC5700942; doi:10.1038/s41598-017-16508-w)
Supplement: Supplementary file 1 — Supplementary Information [file 41598_2017_16508_MOESM1_ESM.pdf]

A novel bi-domain plant defensin MtDef5 with potent broad-spectrum antifungal activity binds to multiple phospholipids and forms oligomers

Kazi T. Islam[1], Siva L S Velivelli[1], R. Howard Berg[1], Blake Oakley[1,2], Dilip M. Shah[1*]

Supplementary information

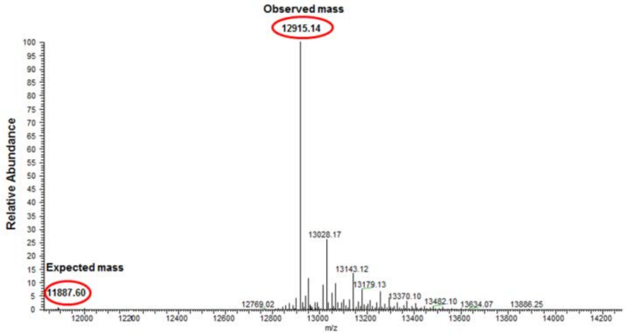

Supplementary Figure S1. ESI-MS analysis of MtDef5 purified from *P. pastoris* cultures. The observed mass of MtDef5 is in agreement with the addition at the amino terminus of this defensin of a nine amino acid sequence EEGVSLEKR derived from the α-factor signal peptide.

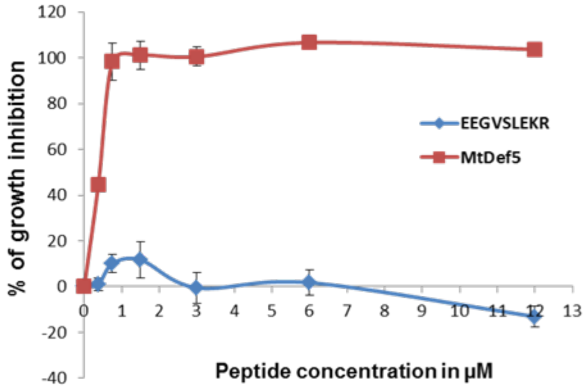

Supplementary Figure S2. Nine amino acid sequence EEGVSLEKR exhibits no antifungal activity against *F. graminearum.* Quantitative assessment of the inhibition of fungal growth of *F. graminearum* was performed at different concentrations of MtDef5 and EEGVSLEKR. Values are means of three replications. Error bars indicate standard deviations.

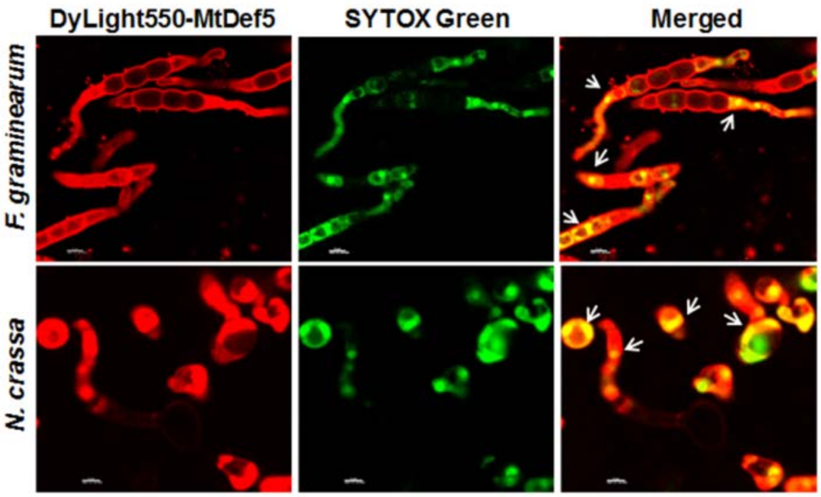

Supplementary Figure S3. MtDef5 is targeted to the nucleus and other subcellular compartments. Dual labeling of germlings of *F. graminearum* and *N. crassa* with 1.5 µM of DyLight550-MtDef5 (red) and nucleic acid-staining dye SYTOX Green (green) shows that DyLight550-MtDef5 is localized in the nuclei as indicated by arrows. MtDef5 is subsequently distributed into the cytoplasm.

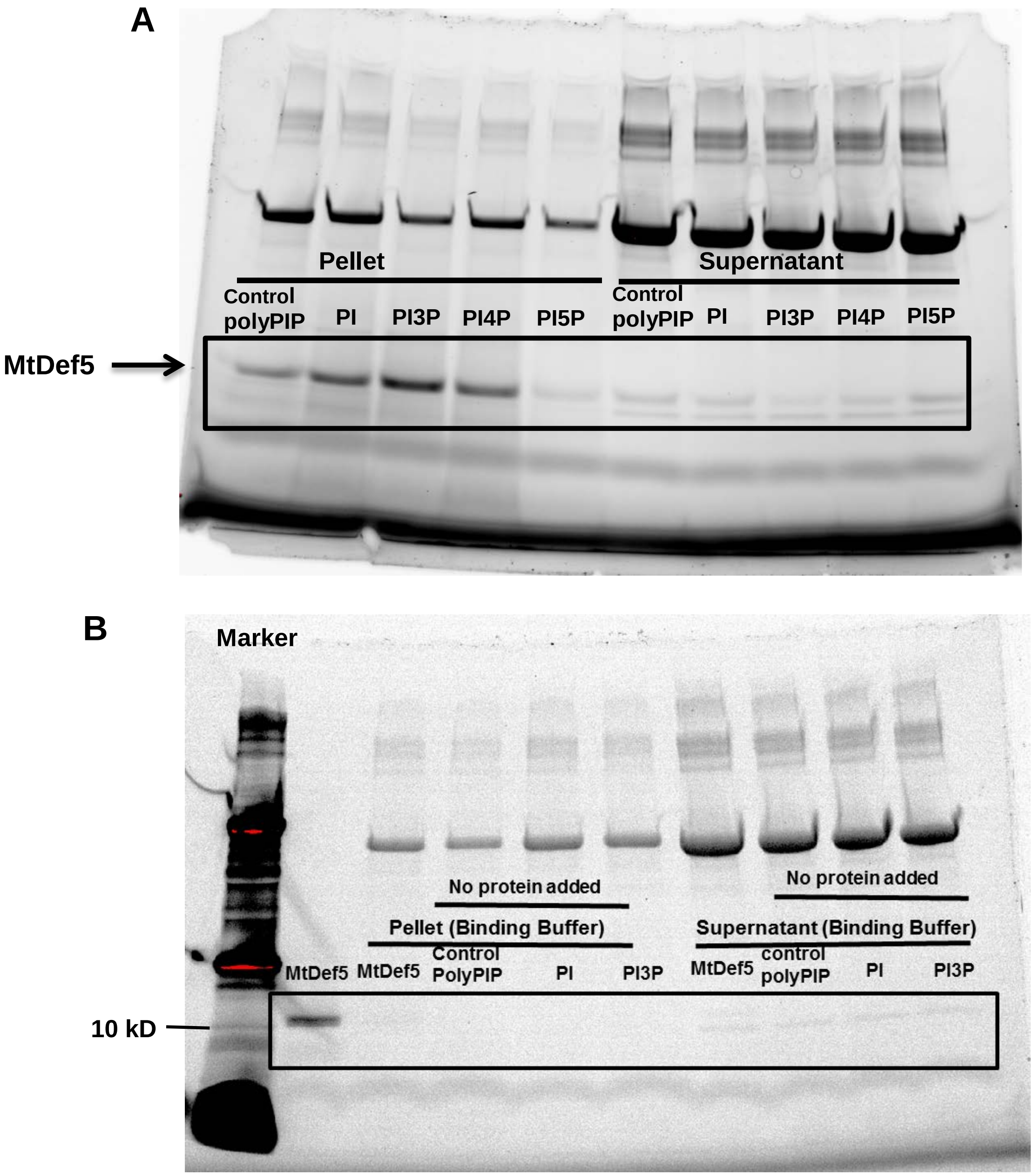

Supplementary Figure S4. PolyPIPosome binding assay. (*A*) MtDef5 binds with higher affinity to PI3P and PI4P as compared to PI5P. (*B*) MtDef5 alone and polyPIPosome with no protein added were used as controls for the PolyPIPosome binding assay to show that binding of MtDef5 to liposomes to phospholipids was specific.

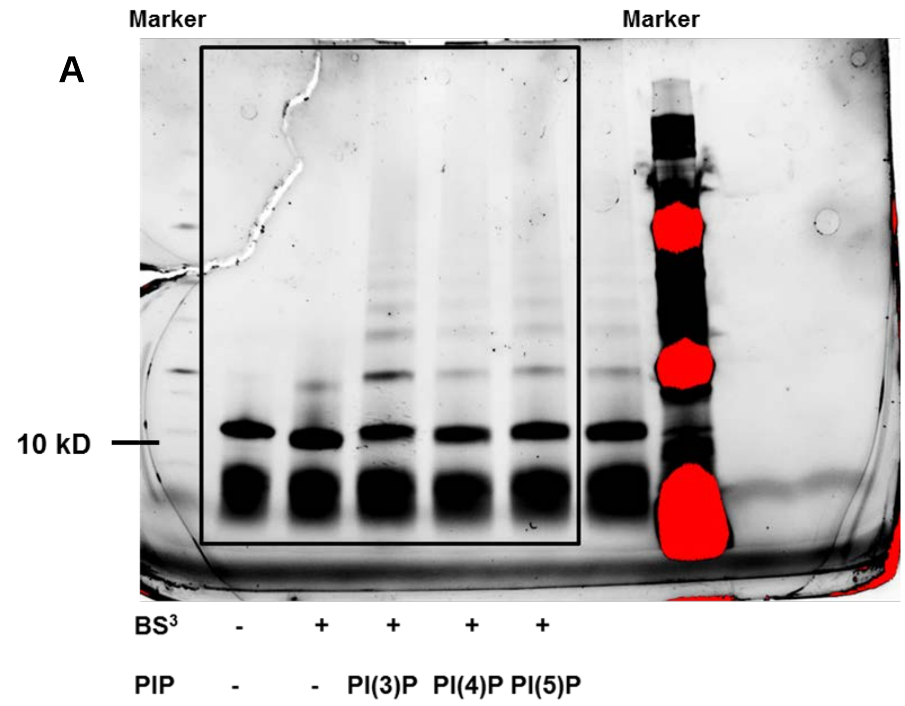

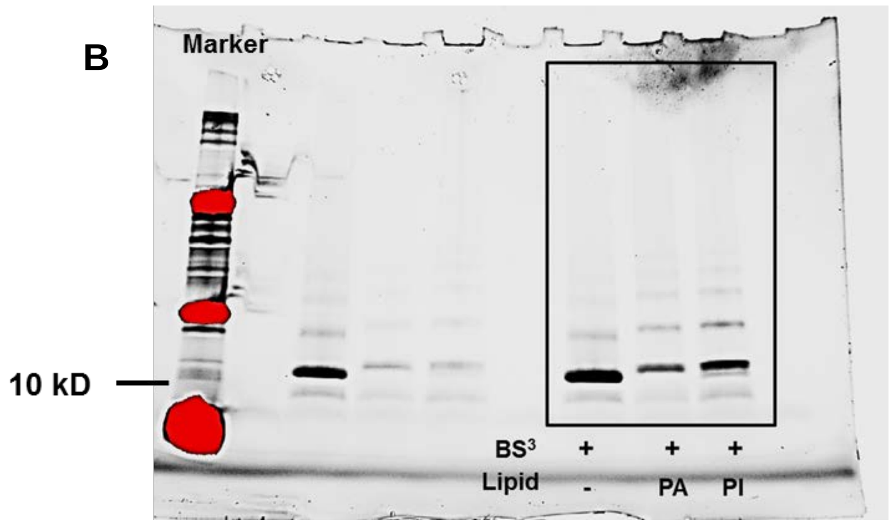

Supplementary Figure S5. MtDef5 forms oligomers in presence of PIP, PI and PA. (*A*) MtDef5 forms higher order oligomers in presence of PI3P, PI4P and PI5P as revealed by a protein cross-linking with BS^3 followed by SDS-PAGE and visualized using a Bio-Rad ChemiDoc XRS+ system as marked in the rectangular box. (*B*) MtDef5 also forms oligomers in presence of PA and PI as marked in the rectangular box.

A

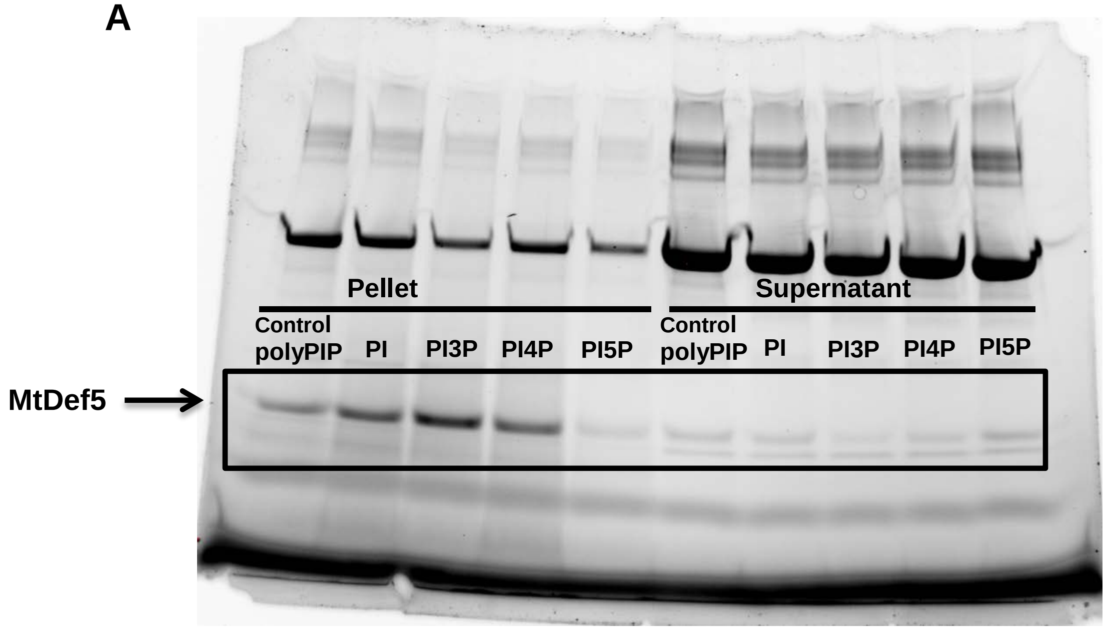
Pellet
Supernatant
Control polyPIP
PI
PI3P
PI4P
PI5P
Control polyPIP
PI
PI3P
PI4P
PI5P
MtDef5

B

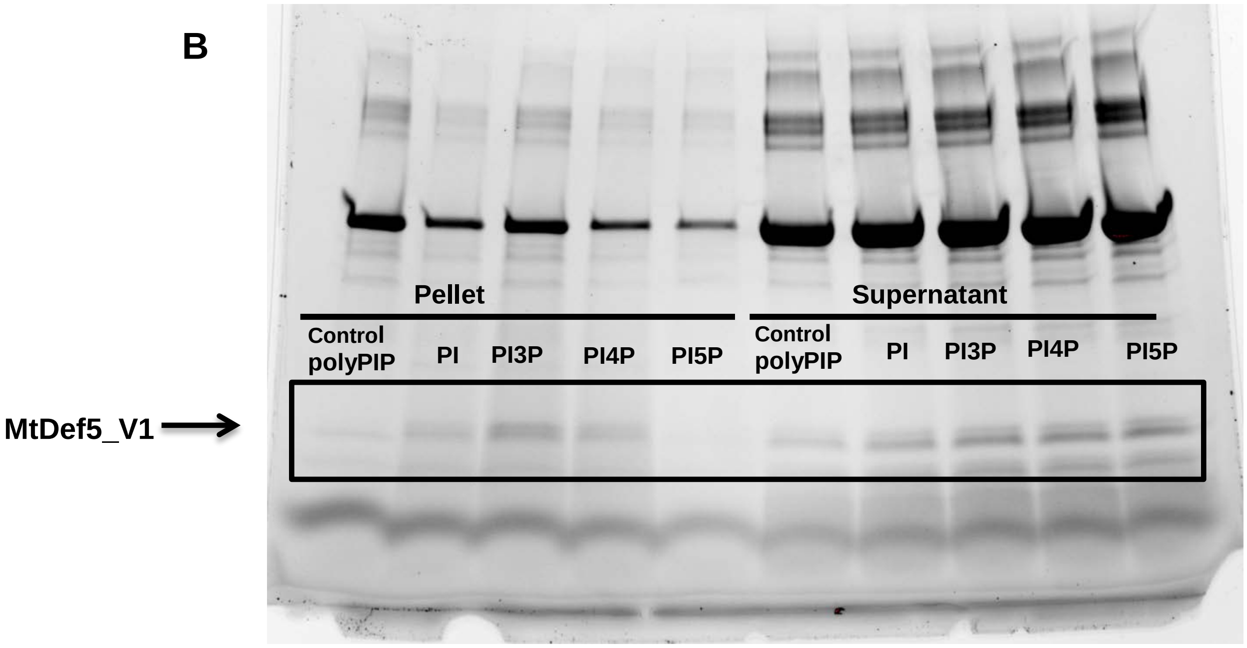
Pellet
Supernatant
Control polyPIP
PI
PI3P
PI4P
PI5P
Control polyPIP
PI
PI3P
PI4P
PI5P
MtDef5_V1

C

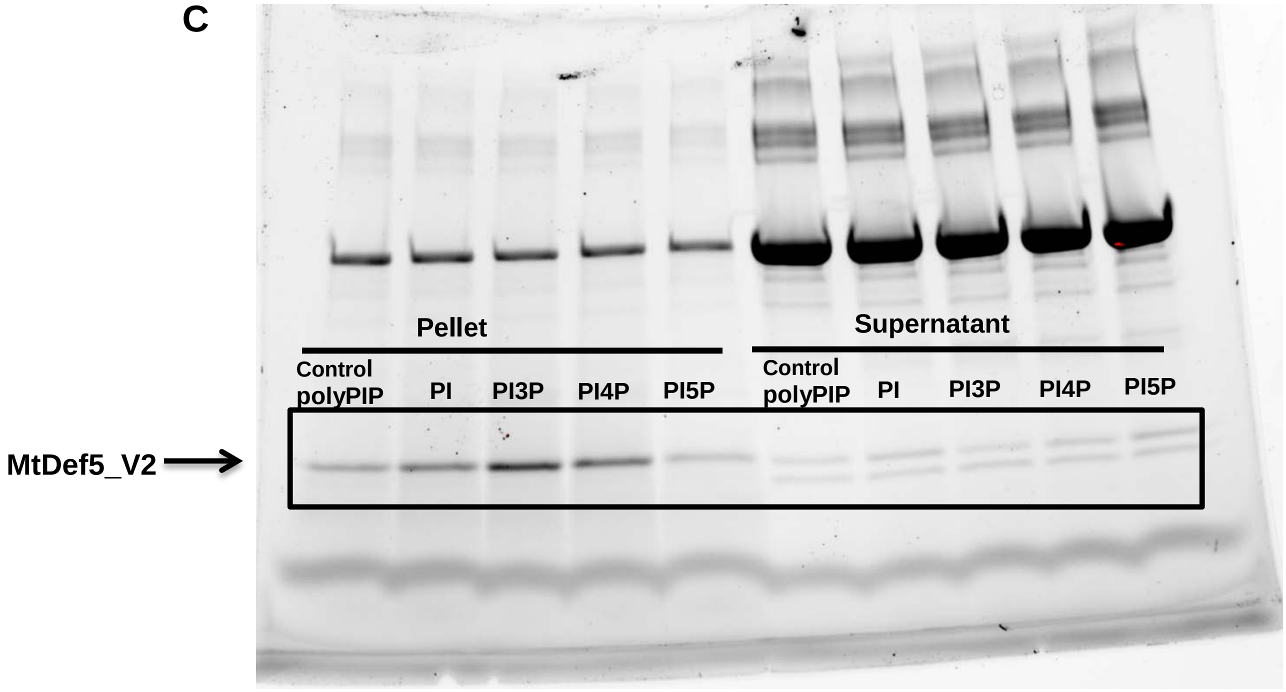
Pellet
Supernatant
Control polyPIP
PI
PI3P
PI4P
PI5P
Control polyPIP
PI
PI3P
PI4P
PI5P
MtDef5_V2

D

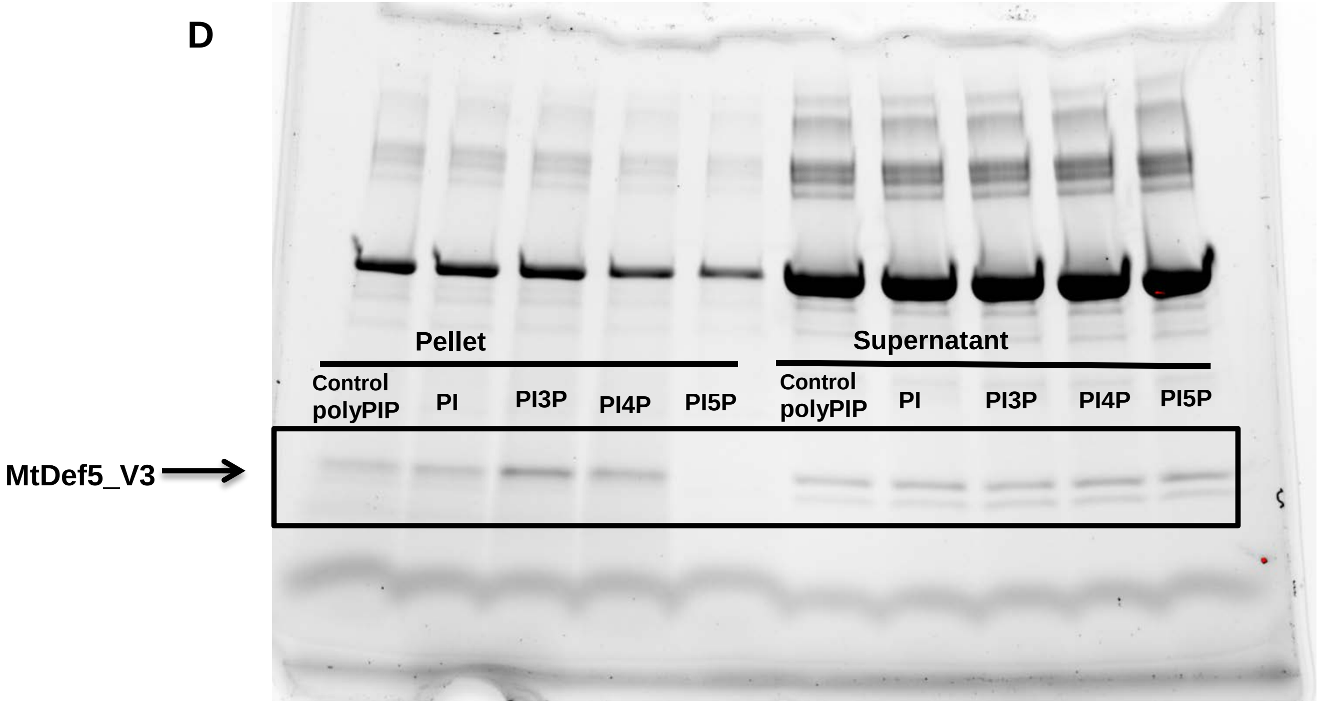
Pellet
Supernatant
Control polyPIP
PI
PI3P
PI4P
PI5P
Control polyPIP
PI
PI3P
PI4P
PI5P
MtDef5_V3

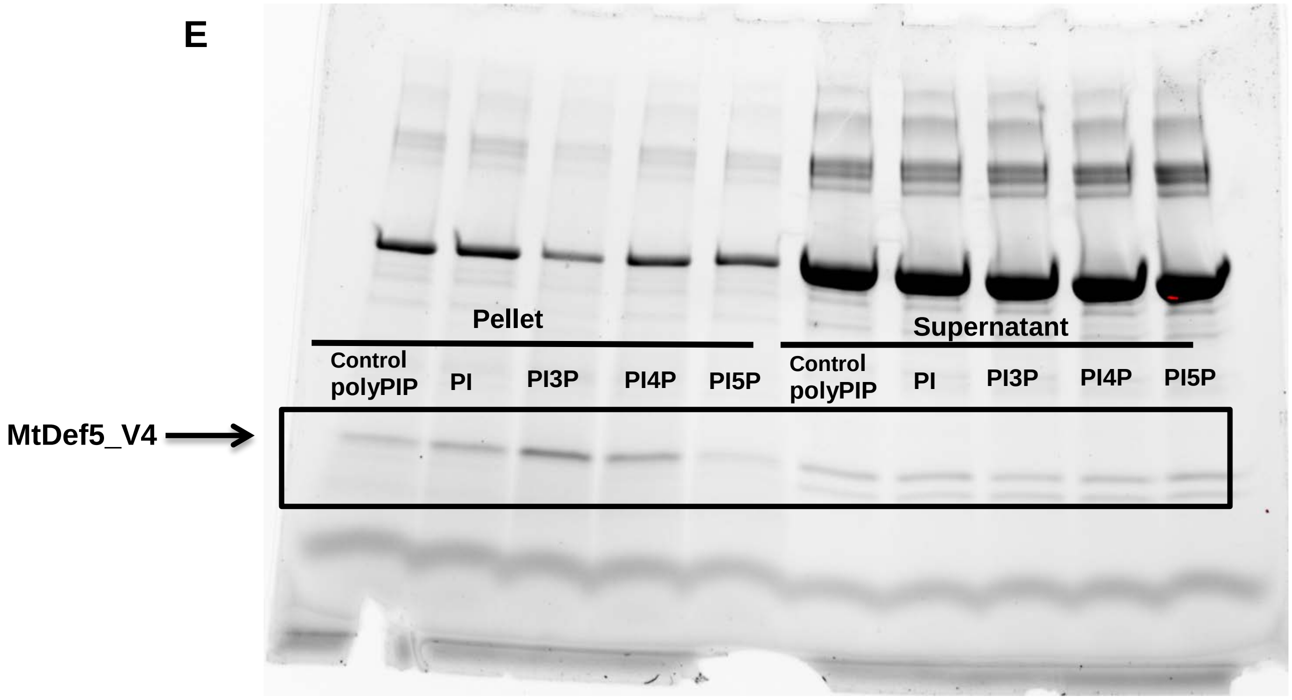

Supplementary Figure S6. PolyPIPosome binding assays of MtDef5 (A) and its γ-core motif variants (B, C, D, E). This assay shows that MtDef5 binds to PI3P and PI4P with higher affinity than PI5P. It also binds to PI.

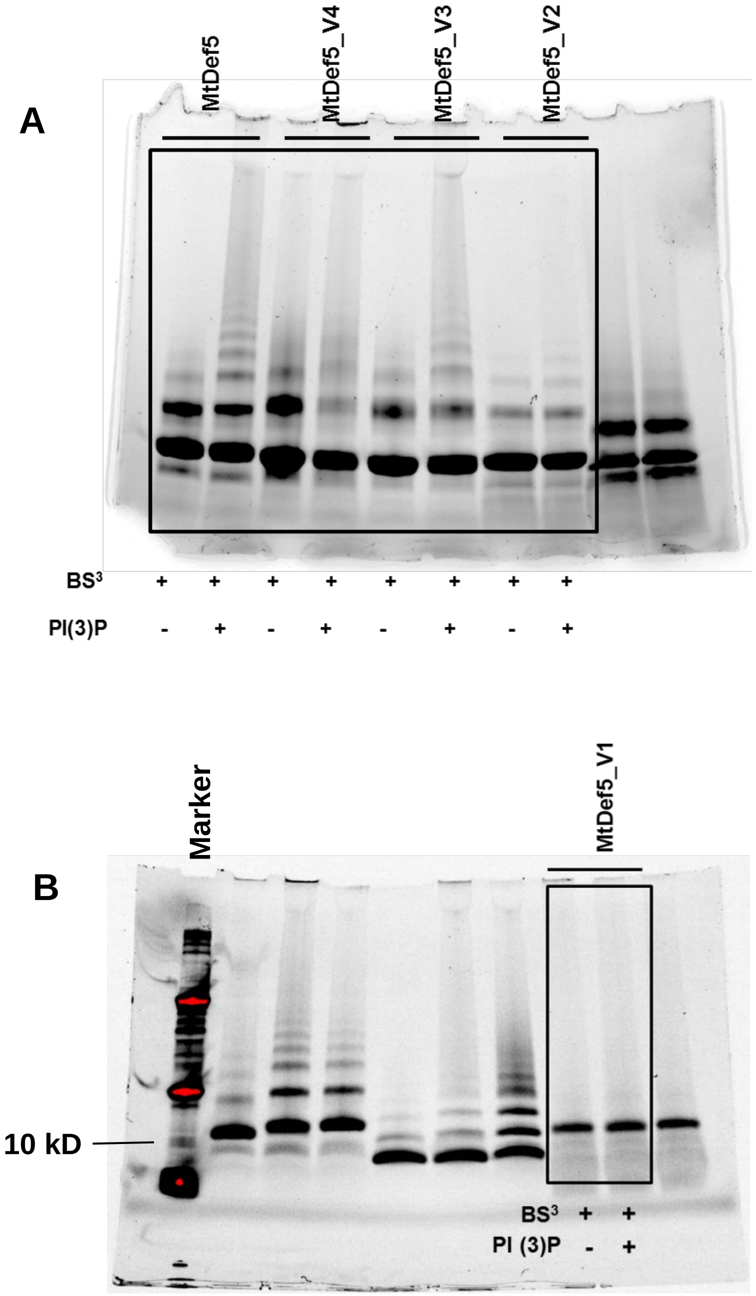

Supplementary Figure S7. γ-core motif sequences of MtDef5 are critical for oligomerization. (*A*) Oligomerization of MtDef5 γ-core motif variants in presence of PI(3)P. (*B*) MtDef5_V1 variant containing the H36A, R37A, H93A and R94A substitutions loses its ability to oligomerize in presence of PI3P as marked in the box.

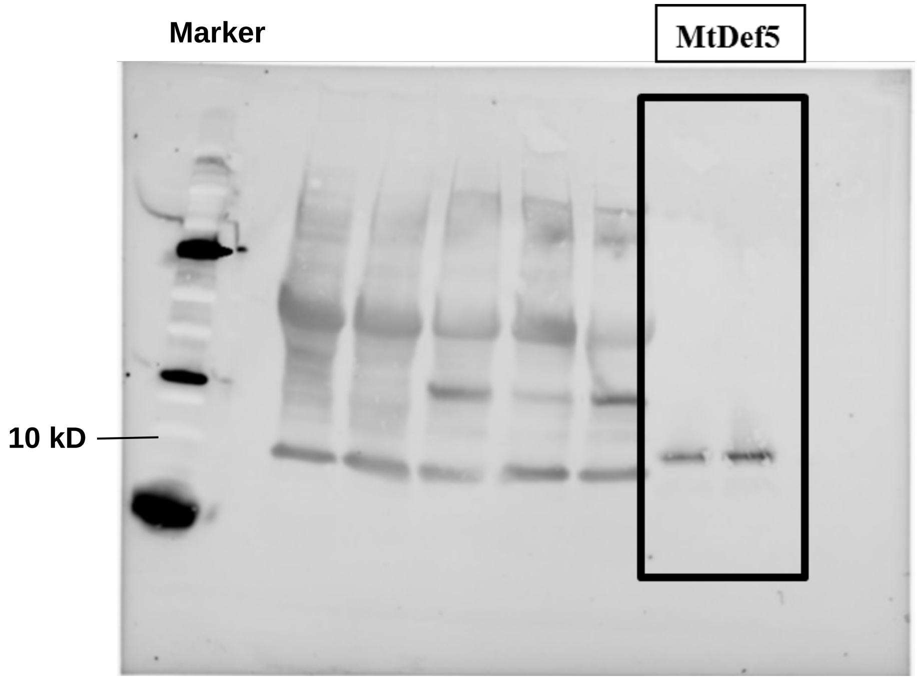

Supplementary Figure S8. Affinity purified MtDef5-derived peptide polyclonal antibody recognizes purified MtDef5 on a Western blot as marked in the box.

Supplementary Table S1. Comparison of MtDef5 characteristics with and without the N-terminal EEGVSLEKR sequence

**Property**[a]	**-EEGVSLEKR**	**+EEGVSLEKR**
Net Charge	+16	+15
Hydrophobic amino acids	39%	40%
Gravy	-0.4794	-0.5465

[a]Calculated values according to Antimicrobial Peptide Database available at http://aps.unmc.edu/AP

Gravy: Grand average of hydropathy index.

Supplementary Table S2. *In vitro* antifungal activity of MtDef5 and its two domains against *F. graminearum*

	**IC_{50} (μM)**	**MIC (μM)**
MtDef5A	0.75-1.0	1.5-3.0
MtDef5B	0.5-0.75	1.0-1.5
MtDef5A+5B	0.75-1.0	1.5
MtDef5	0.25-0.30	0.7-0.75

Supplementary Video Legend

Supplementary Video S1. MtDef5 internalization into cells of *F. graminearum*. Time-lapse live cell imaging showing that DyLight550-MtDef5 is internalized into *F. graminearum* cells and diffuses into the cytoplasm. *F. graminearum* cells were co-stained with DyLight550-MtDef5 (red) and FM4-64 (green). The frequency of image capture was 3 min 30 sec and the total period was 3 h and 26 min. Bar = 5 µM.

Supplementary Video S2. Time-lapse live cell imaging of DyLight550-MtDef5 internalization into *N. crassa. N. crassa* cells were co-stained with DyLight550-MtDef5 (red) and FM4-64 (green). Video was recorded for 2 h and 30 min. Bar = 4 µM.

Supplementary Video S3. MtDef5 is transported to nucleus in *F. graminearum.* Time-lapse imaging showing that MtDef5 is targeted to the nuclei of *F. graminearum* cells. *F. graminearum* germlings were co-stained with 1.5 µM of DyLight550-MtDef5 (red) and nuclear-staining dye SYTOX Green (green). The frequency of image capture was 3 min 30 sec and the total period was 2 h and 3 min. Bar = 5 µM.

Supplementary Video S4. MtDef5 is targeted to nucleus of *N. crassa.* Time-lapse imaging showing that MtDef5 is transported to nuclei of *N. crassa* cells. *N. crassa* germlings were co-stained with 1.5 µM of DyLight550-MtDef5 (red) and nuclear-staining dye SYTOX Green (green). The frequency of image capture was 3 min and the total period was 2 h and 18 min. Bar = 3 µM.
